# Next-generation sequencing-based comparative mapping and culture-based screening of bacterial rhizobiome in *Phytophthora capsici*-resistant and susceptible *Piper* species

**DOI:** 10.3389/fmicb.2024.1458454

**Published:** 2024-09-25

**Authors:** A. Hima Parvathy, R. Santhoshkumar, E. V. Soniya

**Affiliations:** ^1^Transdisciplinary Biology, Rajiv Gandhi Centre for Biotechnology, Thiruvananthapuram, India; ^2^Regional Centre for Biotechnology, Faridabad, India

**Keywords:** *Piper nigrum*, *Piper colubrinum*, Rhizosphere microbiome, *Phytophthora* foot rot resistance, *Pseudomonas*, root endophytes

## Abstract

Black pepper (*Piper nigrum* L.), a highly valued spice crop, is economically significant as one of the most widely traded spices in the world. The global yield and quality of black pepper (*Piper nigrum* L.) are affected by foot rot-causing soil-borne oomycete pathogen *Phytophthora capsici*. To gain initial insights toward developing an approach that utilizes microbial genetic resources for controlling foot rot disease in black pepper, we mapped the rhizobiome communities in susceptible Piper nigrum L. and wild-resistant *Piper colubrinum*. The analysis showed compositional differences in the rhizobiome of two *Piper* species, which revealed higher diversity and the presence of more differentially abundant genera in *P. colubrinum*. Furthermore, *P. colubrinum* rhizobiome had a significantly higher abundance of known anti-oomycete genera, such as *Pseudomonas*, and a higher differential abundance of *Janthinobacterium*, *Variovorax*, and *Comamonas*, indicating their probable contribution to pathogen resistance. Predictive functional profiling in *P. colubrinum* rhizobiome showed highly enriched functional gene orthologs (KOs), particularly chemotaxis proteins, osmoprotectants, and other transport systems that aid in pathogen resistance. Similarly, pathways such as phenylpropanoid biosynthesis and other antimicrobial synthesis were enriched in *P. colubrinum* rhizobiome. The culturable diversity of the resistant root endosphere, which harbors efficient biocontrol agents such as *Pseudomonas*, strengthens the possible role of root microbiome in conferring resistance against soil-borne pathogens. Our results depicted a clear distinction in the rhizobiome architecture of resistant and susceptible *Piper* spp., suggesting its influence in recruiting bacterial communities that probably contribute to pathogen resistance.

## 1 Introduction

Plants have the potential to support specific microbial communities associated with their habitat and requirements. These microbial communities are either specific to the host plant species or its genotype showing temporal as well as geospatial variation (Barraza et al., 2020). The rhizosphere is the part of the soil that is strongly attached to the root system of the host plant, under its direct influence. The rhizobiome, i.e., the microbiome within the root endosphere along with the rhizosphere microbiome, significantly contributes to host health by promoting growth, providing essential nutrients, and inducing stress tolerance as well as pathogen resistance (Poudel et al., 2019; Trivedi et al., 2020). Essentially rhizobiome microbial community could be formed in three ways wherein there is initial colonization of a subset of bulk soil community in the rhizosphere followed by adherence and colonization of a part of this community onto the root surface to form so-called rhizoplane communities, finally, some of these microbes can also enter inside the root and colonize to become endophytic microbial community (Vukanti, 2020; Santoyo et al., 2021; Escudero-Martinez et al., 2022). Several studies have demonstrated the symbiotic association of bacteria in the rhizobiome of plant hosts, complementing to uptake of nutrients from the soil and providing resistance to biotic and abiotic stressors, and in turn, getting nutritional benefits (Vukanti, 2020; Santoyo et al., 2021). Although there is an interplay of multiple factors that control the assembly of bacterial rhizobiome, in general, the type of plant species is the key element that influences the community structure of bacteria in rhizobiome (Berg and Smalla, 2009; Wu et al., 2018). Bacterial rhizobiome establishes complex ecological network associations based on the selective effects exerted by the host plant, which might be independent of other environmental factors. This leads to clear differences in the community composition of the bulk soil when compared to the rhizosphere environment (Bulgarelli et al., 2015; Walters et al., 2018; Rodriguez et al., 2019). Understanding the bacterial community structure and function in rhizobiome specific to the host plant species encompasses the microbial component in sustainable agriculture, which is critical for amending farming practices that lead to optimized plant growth and yield. The disease resistance studies reveal that the integration of meta-omics techniques such as metagenomics can help to initiate further deeper research utilizing advanced methodologies such as microbiome engineering, metabolic engineering, and genome editing (Wille et al., 2021).

Black pepper (*Piper nigrum* L.) belongs to the family Piperaceae, dried fruits of which are used as spices and seasoning. In addition to this, they also contain antioxidant and antimicrobial terpenoids making them an ineluctable component in medicine, healthcare, and food processing (Salehi et al., 2019; Takooree et al., 2019). The cultivation of black pepper is greatly challenged by foot rot disease caused by the oomycete pathogen *Phytophthora capsici* Leonian (*P. capsici*), which persists in soil as resilient zoospores and spreads via irrigation water (Lamour and Hausbeck, 2003; Bhai et al., 2007). The wild *Piper* species, *Piper colubrinum* L. exhibits resistance against *P. capsici* foot rot disease and houses multiple resistance traits, therefore presumes high significance in biotechnological and nanotechnological interventions (Vindran and Remashree, 1998; Varma et al., 2009; Santhoshkumar et al., 2021). Belowground microbial communities are predominantly shaped by plant species, with a stronger influence observed in the rhizosphere bacterial communities and recent studies suggest that bacterial communities may protect host plants from pathogen infection by exerting positive effects on the rhizobiome compartments favoring beneficial microbes (Wu et al., 2018). The growing body of research shows that *Phytophthora* disease resistance is conferred by the microbiome which resides within and in the root vicinity. This indicates that there might be differences in the rhizobiome community composition of wild and cultivated *Piper* species that may influence the disease resistance. Prior studies have demonstrated that this influence exists in other crops plants such as cotton (Qiao QingHua et al., 2017), *Phaseolus vulgaris* (Pérez-Jaramillo et al., 2017), wheat (Quiza et al., 2023). However, the bacterial rhizobiome structure of the *P. colubrinum* remains unknown. Moreover, none of the studies have compared bacterial community composition in wild and cultivated *Piper* in specific locations, which can reveal significant information regarding bacterial communities that may contribute to pathogen resistance. Hence, in this study, we mapped and compared the bacterial community structure and predicted function associated with the rhizobiome of black pepper *P. nigrum* which is susceptible to *P. capsici* infection and *P. colubrinum*, which is known for its resistance to this pathogen using high-throughput 16S ribosomal RNA (16S rRNA) gene-based amplicon sequencing. Both were grown under uniform conditions at the field level and bulk soil was also included for comparative analysis. This study is the first of its kind on black pepper, where the taxonomic composition, abundance, and diversity along with predicted functions of bacterial rhizobiome in wild and cultivated *Piper* are compared to bulk soil. Our study will provide primary insights into the influence of plant species on bacterial rhizobiome diversity in wild (disease resistant) and cultivated (disease susceptible) *Piper* species, which will significantly contribute to pathogen management and sustainable black pepper production.

Along with 16S rRNA-based screening, the study also reports on the culture-based isolation of the abundant bacterial endophytes from the rhizobiome of resistant *Piper colubrinum*, its phylogenetic classification, plant growth promotion, and biocontrol potential assays. We were able to isolate the abundant genus *Pseudomonas* from the root endosphere of *P. colubrinum*, which was able to inhibit the growth of *Phytophthora capsici in vitro.* The 16S rRNA-based metagenomic sequencing and the culturable data shed light on the fact that the resistant rhizobiome harbors bacterial genera with biocontrol potential which may confer disease resistance to the resistant plant species.

## 2 Materials and methods

### 2.1 Study site and sample collection

The soil samples were collected from two sites in the major black pepper growing tracts of the Western Ghats in Idukki district of Kerala, India (9°58′55.2”N 76°52′26.4″ E, 9° 58′ 54.1524” N 76° 52′ 25.356″ E, 600 m above mean sea level) during the south-west monsoon period in July 2022, when there is a higher chance of quick wilt incidence in black pepper. The average annual precipitation and temperature are 2345.90 mm and 23°C, respectively. The sampling site soil is classified as lateritic (Supplementary Figure S1) (Kumar et al., 2021). At each site, nine plants from the wild (*Piper colubrinum*) and cultivated (*Piper nigrum*) black pepper were selected for the rhizobiome collection. Each replicate was obtained by pooling together a set of 3 plants, totaling 18 soil samples including bulk soil (BS = 3 replicates X 2 sites; rhizosphere soil =3 replicatesX2 plant speciesX2sites) (Supplementary Table S1). Furthermore, 12 samples (3X2X2) belonging to the rhizobiome were processed in the laboratory to obtain root endosphere samples. Bulk soil was collected in soil cores approximately 23–30 cm away from the base of the plant. For rhizobiome soil collection, approximately 4–6 roots of 9–12 cm in length were procured from each plant using sterile pruning shears (Supplementary Figure S2). The roots along with the rhizosphere soil adhered to it were kept in a 50 mL tube with autoclaved phosphate buffer (6.35 g/L of NaH_2_PO_4_, 8.5 g/L of Na_2_HPO_4_, pH = 6.5) and trimmed as necessary (McPherson et al., 2018). All samples were transported to the laboratory in iceboxes (4°C) and were sorted for downstream processing immediately to retain sample integrity and prevent any changes in microbial composition.

### 2.2 Processing of rhizosphere and root samples

The rhizosphere soil was separated by vortexing, followed by the sequential sonication of the roots with a set of predetermined parameters (60 s, 60 s, and 10 min) with an output power of 70 W and frequency of 42 kHz (White et al., 2015). This technique gently removes the rhizosphere soil and bacterial biofilms without damaging the plant roots and allows the isolation of microbial communities that exhibit different degrees of association with the root system. The roots, devoid of any soil particles after the sonication step, were segregated and transferred to tubes with fresh autoclaved phosphate buffer (pH = 6.5). Surface sterilization was carried out by treating with 50% sodium hypochlorite for 2 min with shaking, followed by 70% ethanol for an additional 30–60s. To remove any lingering chemicals, the samples were rinsed three times with sterile ultrapure water. Later, the roots were macerated and used for deoxyribonucleic acid (DNA) isolation (McPherson et al., 2018).

### 2.3 DNA extraction, nested PCR, and Illumina sequencing

Metagenomic DNA extraction from the samples was carried out using the DNeasy Power Soil Pro Kit (Qiagen, Germany) according to the manufacturer’s protocol. DNA quality was assessed using the Qubit assay (Invitrogen, ds High sensitivity Assay Cat# Q32854) and agarose gel electrophoresis. Using a nested polymerase chain reaction (PCR) approach, the metagenomic DNA was first amplified using primers 16SF: 5’-AGAGTTTGATCCTGGCTCAG’-3, 16SR: 5’-GGTTACCTTGTTACGACTT’-3 targeting the 16S region (1500 bp). Later, the V3–V4 region (460 bp) was amplified using the primers V3–V4F: 5’-CCTACGGGNGGCWGCAG’-3 and V3–V4R: 5’-GACTACHVGGGTATCTAATCC’-3. The NEBNext Ultra Library Prep Kit for Illumina (NEB, Cat# E7370L) was utilized for the library preparation and the sequencing was carried out in an Illumina MiSeq (Illumina Inc., United States) platform at MedGenome Labs Pvt. Ltd. (Bengaluru, India) as described by Caporaso et al. (2012).

### 2.4 Sequence data processing and statistical analysis

The read quality of the raw data obtained from the sequencing provider was analyzed using FastQC version 0.12.1 (Andrews, 2010), which is efficient in spotting potential problems with high-throughput sequencing data. This was followed by primer and adapter trimming using Trimmomatic 0.39 (Bolger et al., 2014) in the paired-end mode to remove low-quality sequences (Phred quality score – Q-score < 20). The Q-score measures the base calling accuracy in the Illumina sequencing. Selecting a Q-score of greater than 20 would reduce less errors in the obtained reads. The paired-end reads were then overlapped and stitched to form longer reads greater than 400 bp using the NG-merge software 0.3, which corrects errors and ambiguous bases better than other merging programs (Gaspar, 2018). The stitched reads were further processed using the *DADA2* pipeline (version 1.16) (Callahan et al., 2016) with default parameters. Further analysis methods were adopted from recent microbiome studies (Hirpara et al., 2024; Nair et al., 2024). Briefly, read quality and error rate correction were performed on single long stitched reads, followed by chimera removal to obtain amplicon sequence variants (ASVs). The ASVs were assigned taxonomy using the naive Bayesian classifier method implemented in *DADA2*, with a sequence similarity of 97% against the Silva 138.1 prokaryotic small subunit (SSU) taxonomic training dataset. Further analysis was performed using various data analysis packages in R (version 4.1.2) (R Core Team, 2013). The phyloseq object was constructed from the processed ASV table, taxa table, sample metadata, and tree file using *phyloseq* v.1.38.0 (McMurdie and Holmes, 2013). Later, in the phyloseq object, the reads were filtered to remove mitochondrial and chloroplast-derived sequences. The reads were then rarefied to an even sequencing depth to normalize library size differences. Statistical analyses and plotting were conducted using the *microeco* package (version 0.19.0) in R (Liu et al., 2021). For alpha diversity, beta diversity, and all taxonomy-associated plots, significant differences were tested using the Wilcoxon rank-sum test and permutational multivariate analysis of variance (PERMANOVA), with *p*-value correction by FALSE DSCOVERY RATE (FDR) (Benjamini and Hochberg, 1995). The functional prediction was carried out using the R package *Tax4Fun2*, which is memory efficient in predicting habitat-specific functional profiles and has higher accuracy than PICRUSt and Tax4Fun (Wemheuer et al., 2020).

### 2.5 Isolation of abundant bacterial endophytes from the roots of *Piper colubrinum*

The surface sterilized root tissues of *Piper colubrinum* were macerated aseptically in 1 mL of phosphate-buffered saline solution (pH 7.4) with mortar and pestle. The diluted (10^−1^) macerate (0.1 mL) was spread on nutrient agar and LB agar plates. The agar plates were incubated at 28°C for 2–3 days. Morphologically different bacterial colonies that were abundant in the plates were subcultured and pure cultures were maintained for further studies (Kollakkodan et al., 2017).

### 2.6 *In vitro Phytophthora* antagonism of the bacterial isolates

The bacterial isolates were screened for their potential to inhibit *Phytophthora capsici* growth on potato dextrose agar using a dual culture plate assay. *P. capsici* mycelial plug was kept at the center of PDA plates and fresh bacterial cultures were streaked on either side of the mycelial plug equidistantly after 2 days of mycelial growth. The plates were incubated at 28°C for 4–5 days to assess the ability to inhibit the mycelial growth based on the inhibition zone (Aravind et al., 2009; Hyder et al., 2020).

### 2.7 Molecular identification of the potential bacterial isolates

Genomic DNA from overnight-grown bacterial cultures was isolated using a Macherey-Nagel Bacterial DNA isolation kit () as per the manufacturer’s instructions. The 16srRNA gene was amplified using the primers 1-27F (GAGAGTTTGATCCTGGCTCAG) and 1495R (CTACGGCTACCTGTTACGA) manufactured by Sigma Aldrich, Germany (Hongoh et al., 2003). PCR was in 10 μL total volume 1X initiation cycle at 95°C for 5 min, 30X denaturation cycles at 95°C for 30 s, 30X primer annealing cycles at 55°C for 30 s, 30X extension cycles at 72°C for 2 min, and a 1X elongation cycle at 72°C for 5 min followed by termination at 4°C. Sequencing PCR of the PCR product was conducted using Big Dye Terminator v3.1 Cycle sequencing Kit (Applied Biosystems, United States) following the manufacturer’s protocol. The sequencing of the purified products was conducted using the Sanger method in a 3730xL DNA Analyzer (96 capillary high-throughput DNA Sequencer) at the Genomics facility, Rajiv Gandhi Center for Biotechnology, Kerala, India.

### 2.8 Phylogenetic analysis

The sequences were used to identify closely related organisms in the National Center for Biotechnology Information (NCBI) BLASTn in the GenBank database^1^ and EzTaxon Database (Chun et al., 2007). The phylogenetic tree was constructed using MEGA (version 11.0.13) using multiple sequence alignment by ClustalW and the neighbor-joining method was used with a bootstrap value of 1,000.

### 2.9 Qualitative plant growth promotion assays of potential bacterial isolates

The following plant growth promotion traits of the bacteria with biocontrol potential were evaluated: indole acetic acid (IAA) production, siderophore production, hydrogen cyanide (HCN) production, and β-1-4-glucanse activity. The methodology followed is briefly explained below:

#### 2.9.1 IAA production

The isolates were incubated in 10 mL of nutrient broth containing 0.2% (v/v) L-tryptophan at 28°C for 10 days. Later, the culture was centrifuged for 20 min at 10000 rpm to check for the presence of IAA in the supernatant (Bric et al., 1991). After adding 2 mL of Salkowski reagent (35% HClO_4_, 0.5 M FeCl_3_) to 1 mL of culture supernatant, the tubes were incubated at 28 ± 2°C in the dark for 30 min. A color change from yellow to pink indicates the presence of IAA in the medium.

#### 2.9.2 Siderophore production

Blue Chrome Azur S agar medium was used to test for siderophore production (Louden et al., 2011). Briefly, Blue Agar-CAS medium was made with 100 mL of blue dye, 30 mL of filter-sterilized 10% Casamino acid solution, 10 mL of 20% glucose, and autoclaved MM9 salt medium and agar [added with 32.24 g piperazine-N, N0-bis2-ethane sulfonic acid (PIPES) at pH 6]. Overnight cultures of the isolates were streaked on the plates. A color change of the medium around the bacterial growth indicates siderophore production.

#### 2.9.3 HCN production

Isolates were inoculated on nutrient agar plates supplemented with glycine at a concentration of 4.4 g L^−1^. A sterile filter paper (Whatman No.1) saturated with picric acid (0.5%) and sodium carbonate (2%) was placed in the lid of the Petri plate facing the agar inoculated with the fresh cultures of the isolates. The color change of the filter paper from yellow to orange-red indicates HCN production (Lorck, 1948).

#### 2.9.4 β-1-4-glucanse activity

β-1-4-glucanse activity was tested following the protocol of (Hendricks et al., 1995). Spot inoculation of the bacterial isolates was conducted on carboxymethyl cellulose (CMC) agar with Congo red as an indicator. A halo around the bacterial growth indicates positive for β-1-4-glucanse production.

## 3 Results

### 3.1 Bacterial abundance and diversity in rhizobiome of *Piper* species

**Figure 1:**
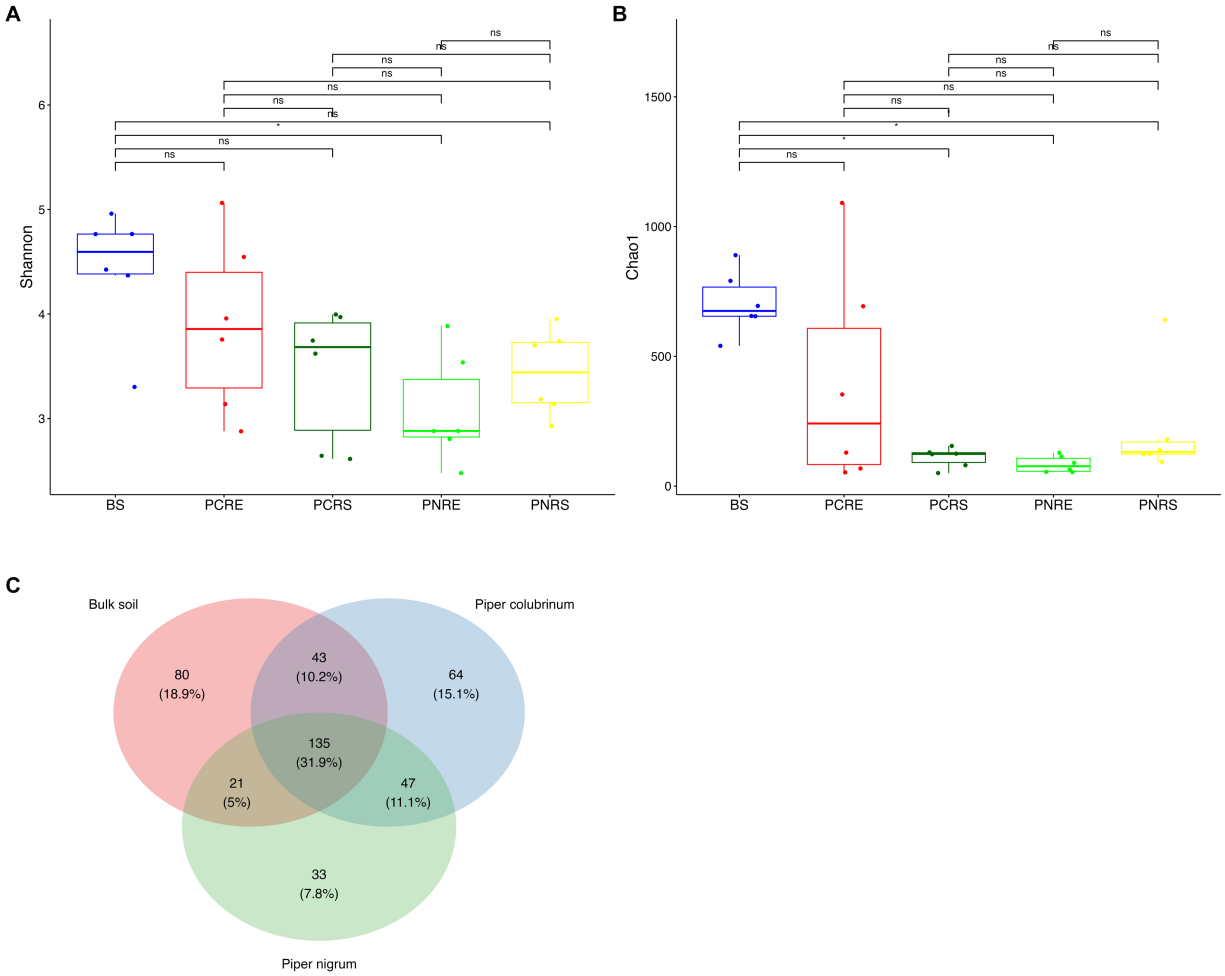
Box plots showing the distribution of alpha diversity indices of bacterial amplicon sequence variants (ASVs) **(A)** Shannon diversity index and **(B)** Chao 1 index (*P<0.05; Wilcoxon test); **(C)** Venn diagram illustrating the total number and the shared genera in bulk soil, *Piper colubrinum* and *Piper nigrum* (BS: Bulk soil; PNRE: *Piper nigrum* root endosphere; PNRS: *Piper nigrum* rhizosphere soil; PCRS: *Piper colubrinum* rhizosphere soil; PCRE: *Piper colubrinum* root endosphere).

A total of 6,088 good-quality bacterial ASVs were retained after filtering across all soil compartments of both the wild-resistant (*P. colubrinum*) and cultivated susceptible (*P. nigrum*) species from the rarefied datasets, which comprised 6,823 reads per sample. Venn diagram indicated the presence of unique and shared bacterial genera among *P. colubrinum*, *P. nigrum*, and bulk soil (Figure 1C). A total of 31.9% of bacterial genera were shared among wild-resistant and cultivated susceptible species and the bulk soil. *P. colubrinum* (10.2%) shared more bacterial genera with bulk soil than *P. nigrum* (5%), whereas both species shared 11.1% of bacterial genera among them. The distribution of unique bacterial genera varied, and it was found to be highest in bulk soil (18.9%), followed by *P. colubrinum* (15.1%) and *P. nigrum* (7.8%) (Figure 1C).

**Figure 2:**
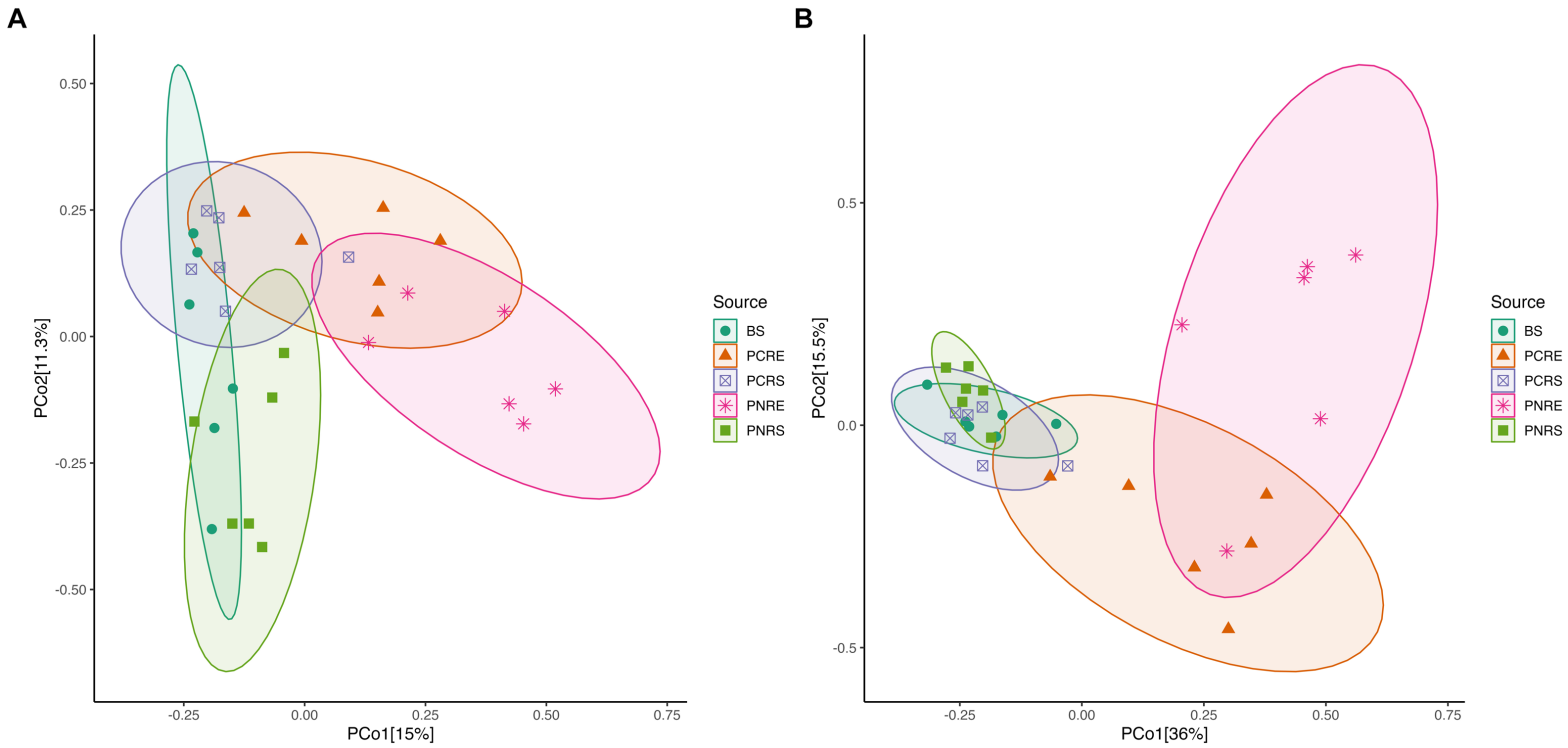
PCoA plot based on Bray Curtis distance among bulk soil, and the root endosphere and rhizosphere soil of *P. nigrum* and *P.colubrinum* at **(A)** the amplicon sequence variant (ASV) level and **(B)** at genus level. (BS: Bulk soil; PNRE: *Piper nigrum* root endosphere; PNRS: *Piper nigrum* rhizosphere soil; PCRS: *Piper colubrinum* rhizosphere soil; PCRE: *Piper colubrinum* root endosphere).

The Shannon and Chao1 indices showed a higher significant (*p < 0.05, Wilcox) alpha diversity in bulk soil compared to the rhizobiome compartments of both plant species. (Figures 1A,B). Considering the overall diversity and richness, *P. colubrinum* rhizobiome had higher bacterial diversity compared to that of *P. nigrum*, even though the differences were not statistically significant (Supplementary Table S2). Furthermore, compositional dissimilarities were assessed using principal coordinate analysis (PCoA) based on the Bray–Curtis distance. The findings exhibited significant variation (PERMANOVA; FDR, *p < 0.05) among the bacterial communities at ASV as well as genus level across bulk soil and rhizobiome of *P. colubrinum* and *P. nigrum* (Table 1). There was an overlap in bacterial ASVs and genera among the rhizosphere and bulk soil compartments, while the endosphere communities remained distinct (Figures 2A,B). These results suggest a marked difference in bacterial community composition among the wild and cultivated *Piper* rhizobiome, with distinct closeness among rhizosphere and bulk soil communities compared to the endosphere.

**Table 1:** Pairwise PERMANOVA statistics show significant differences (**p < 0.01; *p < 0.05) in the composition of bacterial communities assessed using PCoA.

ASV level	Groups	Measure	*F*	*R* ^2^	*p*- value	*p*. adjusted	Significance
1	BS vs. PCRE	Bray	2.8334	0.2208	0.0040	0.0057	**
2	BS vs. PCRS	Bray	2.8166	0.2198	0.0030	0.0057	**
3	BS vs. PNRE	Bray	4.2478	0.2981	0.0030	0.0057	**
4	BS vs. PNRS	Bray	2.8349	0.2209	0.0090	0.0090	**
5	PCRE vs. PCRS	Bray	1.9713	0.1647	0.0080	0.0089	**
6	PCRE vs. PNRE	Bray	2.2269	0.1821	0.0070	0.0088	**
7	PCRE vs. PNRS	Bray	2.9240	0.2262	0.0030	0.0057	**
8	PCRS vs. PNRE	Bray	4.1304	0.2923	0.0020	0.0057	**
9	PCRS vs. PNRS	Bray	2.8822	0.2237	0.0040	0.0057	**
10	PNRE vs. PNRS	Bray	3.7993	0.2753	0.0030	0.0057	**
Genus level							
1	BS vs. PCRE	Bray	6.8592	0.4069	0.0050	0.0075	**
2	BS vs. PCRS	Bray	6.0880	0.3784	0.0020	0.0075	**
3	BS vs. PNRE	Bray	10.1314	0.5033	0.0050	0.0075	**
4	BS vs. PNRS	Bray	6.7883	0.4043	0.0060	0.0075	**
5	PCRE vs. PCRS	Bray	6.2350	0.3840	0.0040	0.0075	**
6	PCRE vs. PNRE	Bray	3.7869	0.2747	0.0080	0.0089	**
7	PCRE vs. PNRS	Bray	8.5513	0.4610	0.0030	0.0075	**
8	PCRS vs. PNRE	Bray	10.0180	0.5004	0.0060	0.0075	**
9	PCRS vs. PNRS	Bray	2.3528	0.1905	0.0160	0.0160	*
10	PNRE vs. PNRS	Bray	11.1141	0.5264	0.0020	0.0075	**

((BS, Bulk soil; PNRE, Piper nigrum root endosphere; PNRS, Piper nigrum rhizosphere soil; PCRS, Piper colubrinum rhizosphere soil; PCRE, Piper colubrinum root endosphere)..

### 3.2 Taxonomic composition of bacterial communities in bulk soil and rhizobiome of wild-resistant and cultivated susceptible *Piper*

The dominant phylum in bulk soil, as well as resistant and susceptible *Piper*, was *Proteobacteria*, constituting 76–93% of the bacterial community, followed by *Firmicutes* and *Bacteriodota*, which contributed an abundance up to 4–22% and 1–6%, respectively. The rhizospheric bacterial communities in *P. colubrinum* predominantly consisted of *Proteobacteria* (92.86%), while *P. nigrum* had them in lower abundance (89.67%), along with *Firmicutes* (4.81%) and *Bacteriodota* (3.98%). In the root endosphere, the abundance proportion of the dominant phyla varied between the two plant species. Abundance was contributed by *Proteobacteria* (76.48%) and *Firmicutes* (22.56%) in *P. nigrum*. In the root endosphere of *P. colubrinum*, the abundance of *Proteobacteria* (93.16%) was higher, whereas *Firmicutes* (4.52%) and *Bacteroidota* (1.07%) were present in lower proportions compared to *P. nigrum* (Supplementary Table S3).

**Figure 3:**
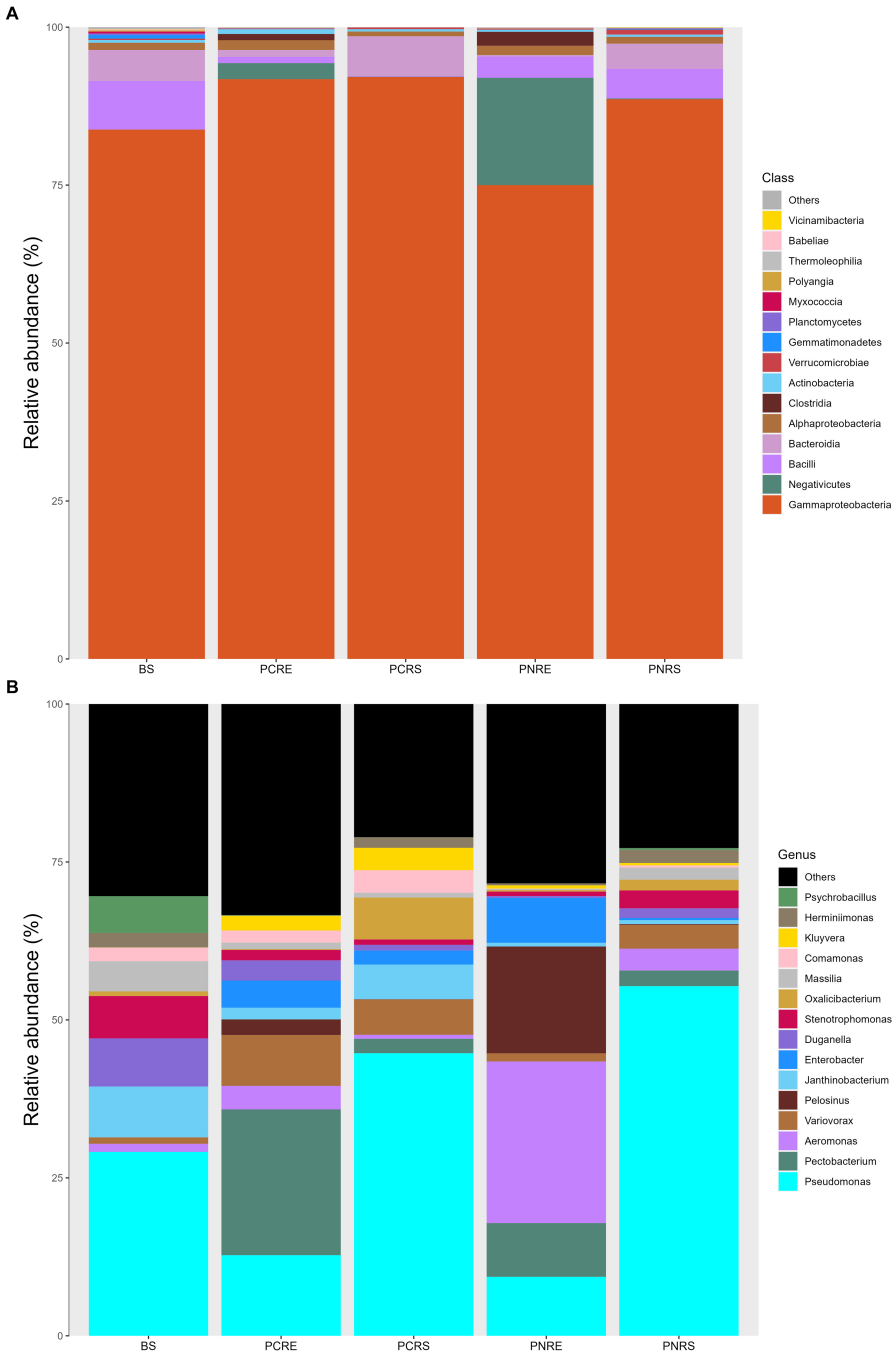
Relative abundance of taxa in percentage at **(A)** Class level and **(B)** Genera level (BS: Bulk soil; PNRE: *Piper nigrum* root endosphere; PNRS: *Piper nigrum* rhizosphere soil; PCRS: *Piper colubrinum* rhizosphere soil; PCRE: *Piper colubrinum* root endosphere).

In phylum *Proteobacteria*, the abundance of class *Gammaproteobacteria* was highest among others in bulk soil and rhizobiome of *P. nigrum* and *P. colubrinum*, with a higher abundance proportion in rhizobiome of *P. colubrinum* (91–92%) than *P. nigrum* (75–88%). Furthermore, classification at class level showed Negativicutes*, Bacilli, and Bacteroidia* as the most abundant members among top 15 in the bulk soil and rhizobiome (Figure 3A). *Negativicutes* showed higher abundance in root endosphere of *P. nigrum* (16.9%) than *P. colubrinum* (2.5%) and *Bacteriodia* was found in higher proportions among the most abundant in rhizosphere of *P. colubrinum* (6.5%) (Supplementary Table S3).

At the genus level, the bacterial community members showed a varied abundance pattern in the bulk soil and rhizobiome. Genus *Pseudomonas* showed higher abundance in the rhizosphere (44–55%), followed by bulk soil (29%) and root endosphere (9–12%). The root endosphere of *P. colubrinum* had a higher percentage (12.75%) of genus *Pseudomonas* than that of *P. nigrum* (9.32%). In root endosphere, genus *Aeromonas* was seen in higher proportion in *P. nigrum* (25.07%) than *P. colubrinum* (3.7%) (Figure 3B). Altogether, these results show clear variation in abundance values of top abundant bacterial members of bulk soil, wild and cultivated *Piper*, with increasing variation in the abundance pattern at lower taxonomic ranks (genus).

### 3.3 Differentially abundant bacterial rhizobiome communities in *Piper* species

**Figure 4:**
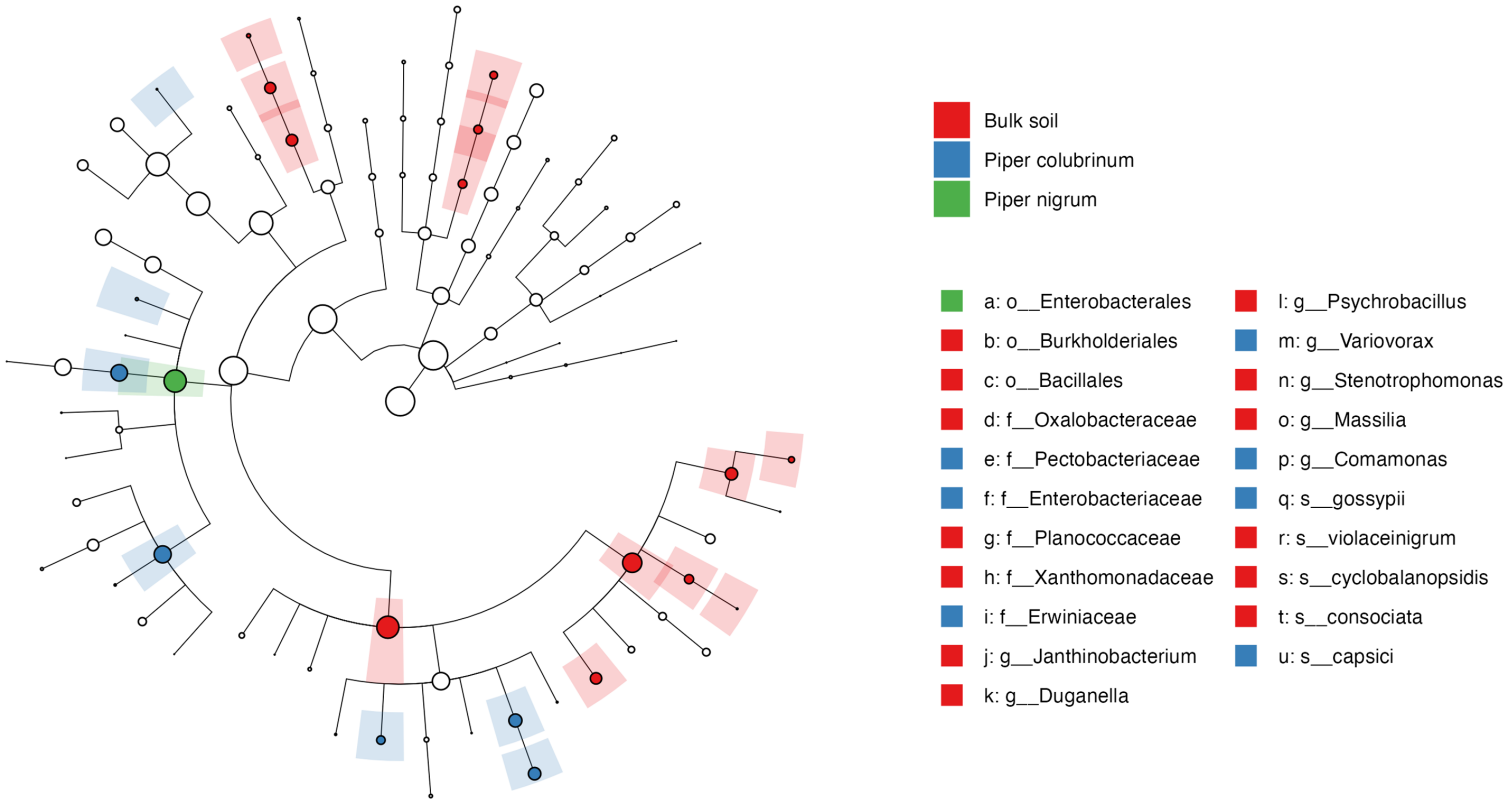
Cladogram of Linear discriminant analysis (LDA) effect size (LEfSe) analysis of microbial abundance in the bulk soil and the hosts.

To further investigate the significance of variation found in the abundance profile of bacterial communities, a differential abundance analysis was performed. The cladogram based on linear discriminant analysis (LDA) effect size (Lefse) analysis of the top 100 most abundant taxa revealed significant differences in the phylogenetic composition of bacterial communities in the rhizobiome of *P. colubrinum* and *P. nigrum*. Twenty-one bacterial clades were found differentially abundant (LDA score > 3.0) among the bulk soil and rhizobiome of *Piper* hosts across all taxonomic ranks, in which resistant *P. colubrinum* rhizobiome had more discriminatory clades than the susceptible *P. nigrum* (Figure 4). These included the genera *Comamonas* and Var*iovorax*, which were significantly higher in the *P. colubrinum* rhizobiome (Kruskal–Wallis test, *p < 0.05), along with few plant growth-promoting bacteria such as *Variovorax gossypii* and *Pseudomonas capsici* (Supplementary Figure S3). Furthermore, differential abundance analysis of the bacterial communities using ANCOMBC-II showed that the relative abundances of a few bacterial genera differed significantly at the genus level between the rhizobiome of resistant *P. colubrinum* and susceptible *P. nigrum* (Supplementary Figure S4). In *P. colubrinum* rhizosphere, a significant difference in abundance was found only in the genus *Janthinobacterium* (*p < 0.05), while other genera such as *Comamonas* and *Variovorax* were differentially abundant but not significant. In *P. nigrum* rhizosphere*, Pseudomonas* showed higher abundance values than *P. colubrinum*; however, the differences were not significant. (Supplementary Figure S4A). In the root endosphere, significant differences were also observed in the abundance values of *Comamonas* (***p < 0.001) in *P. colubrinum* and *Aeromonas* (**p < 0.01) in *P. nigrum* (Supplementary Figure S4B). Conclusively, the analysis suggests phylogenetic distinctness among bacterial communities in both *Piper* species, with significant variation in their abundance profile.

### 3.4 Functional signatures in the rhizobiome of *Piper* species

In metabolic pathways (level 2) predicted using the Kyoto Encyclopedia of Genes and Genomes (KEGG) database, there was a higher abundance of pathway categories such as infectious diseases: bacterial, glycan biosynthesis and metabolism, folding, sorting and degradation, nucleotide metabolism, carbohydrate metabolism, and energy metabolism in the root endosphere of *P. nigrum* compared to *P. colubrinum*, while these differences were not observed in the rhizosphere of these two plants. The metabolic pathway categories such as “Cellular community- prokaryotes” and “Biosynthesis of other secondary metabolites” were found to be highly abundant in *P. colubrinum* compared to *P. nigrum* (Supplementary Figure S5). Furthermore, a cluster analysis was performed for the top 100 KEGG Orthology (KO) functional orthologs among bulk soil, and *Piper* rhizobiome showed a higher abundance of functional orthologs in rhizosphere soil compared to bulk soil and endosphere. KOs such as chemotaxis proteins, osmoprotectants, and other transport systems showed higher abundance in the rhizobiome of resistant *P. colubrinum* compared to susceptible *P. nigrum* (Supplementary Figure S6).

### 3.5 Isolation, identification, and characterization of the abundant biocontrol genera from the resistant root endosphere

**Figure 5:**
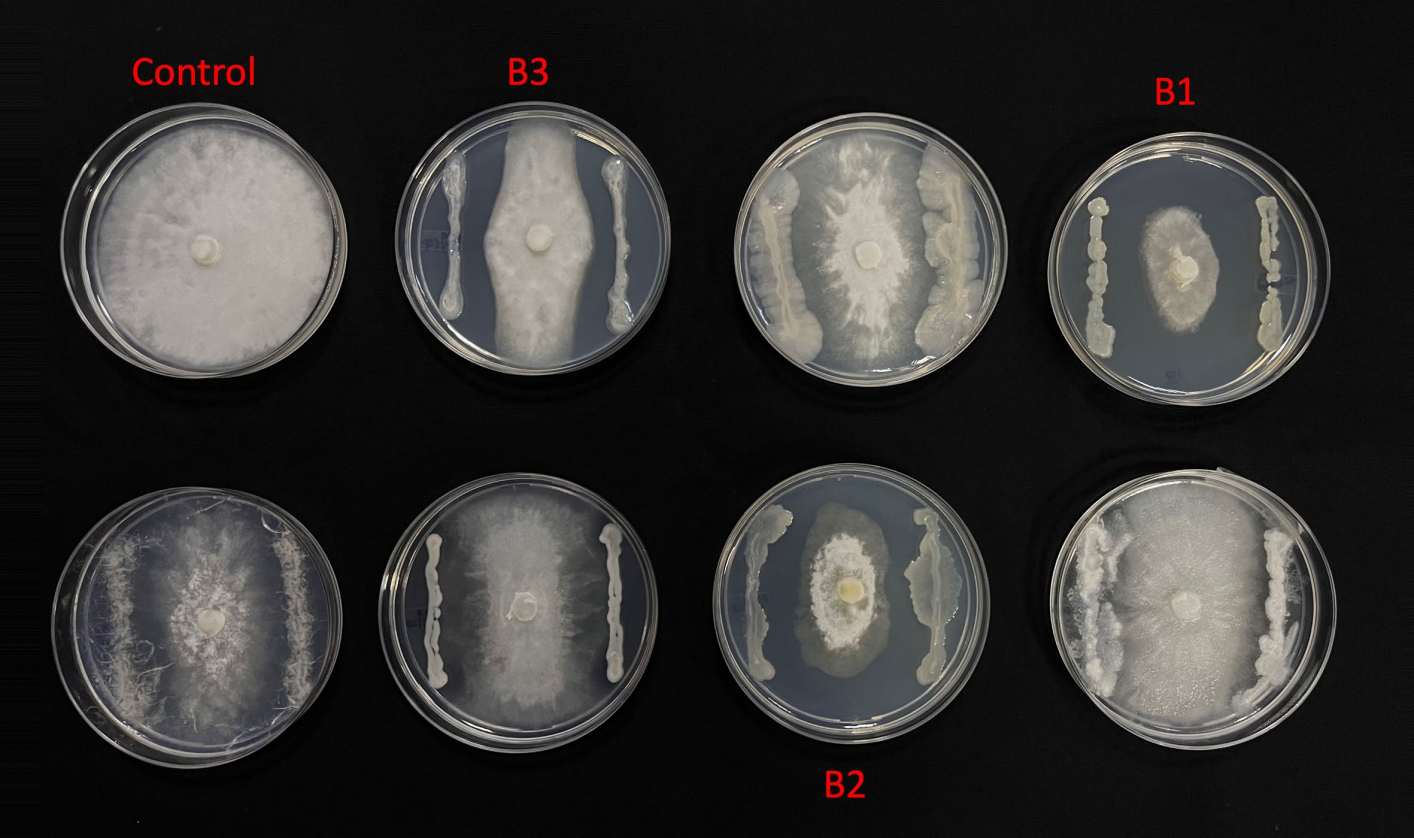
*In vitro* Phytopthora antagonism of bacterial isolates (B1: Pseudomonas aeruginosa; B2: Pseudomonas mosselii; B3: Pseudomonas sichuanensis).

**Figure 6:**
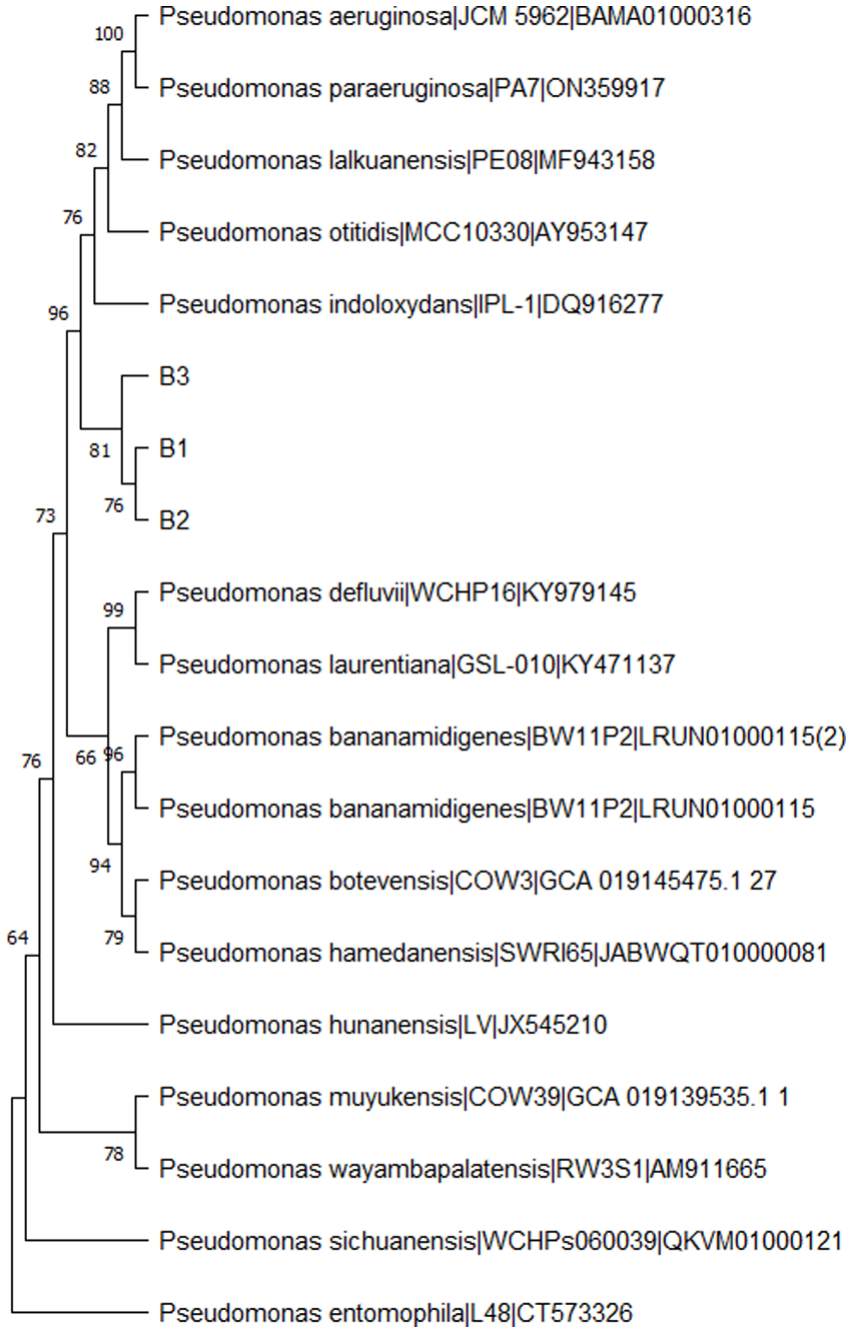
Phylogenetic tree of bacterial isolates (B1: *Pseudomonas aeruginosa*; B2: *Pseudomonas mosselii*; B3: *Pseudomonas sichuanensis*) constructed using MEGA (Version 11.0.13) by neighbour-joining method with a bootstrap value of 1000.

**Figure 7:**
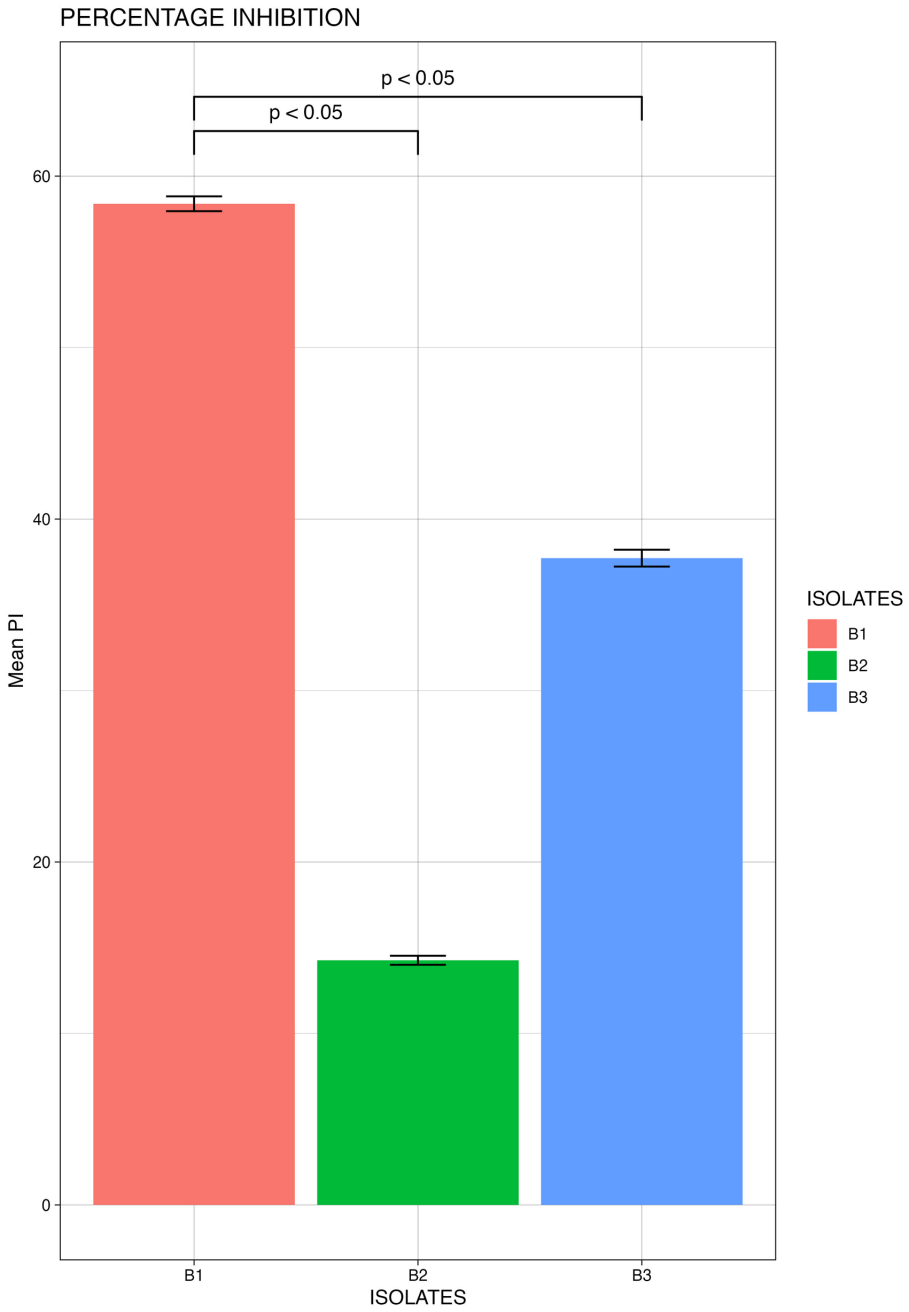
Mean Percentage inhibition P. capsici by the bacterial isolates (B1: *Pseudomonas aeruginosa*; B2: *Pseudomonas mosselii*; B3: *Pseudomonas sichuanensis*) (significance p<0.05; mean of three replicates each n=3).

From the morphologically different colonies isolated, those isolates which exhibited maximum percentage inhibition of the oomycete pathogen *P. capsici* in dual plate assay were selected for characterization. They were further characterized phylogenetically and evaluated for their plant growth promotion traits. Among 12 morphologically different isolates, 3 efficiently inhibited *P. capsici in vitro* (Figure 5), and they were named B1, B2, and B3. The 16S rRNA gene sequencing and blast analysis in EzTaxon and NCBI showed maximum similarity percentage as B1 - *Pseudomonas aeruginosa* (87–89%), B2 - *Pseudomonas bananamidigenes* (91.58%)*/P. mosselii* (94.17%), and B3 - *Pseudomonas sichuanensis* (94.24–94.65%), respectively. The sequences retrieved from EzTaxon and NCBI were used for phylogenetic analysis with MEGA (version 11.0.13). The three isolates (B1, B2, and B3) formed separate clades, indicating that they are different species and they have a paraphyletic relationship with other *Pseudomonas* species (Figure 6). Among the three, B1 exhibited a significantly high percentage inhibition potential against *P. capsici* (Figure 7).

### 3.6 Determination of IAA, HCN, β-1-4-glucanse, and Siderophore production

**Figure 8:**
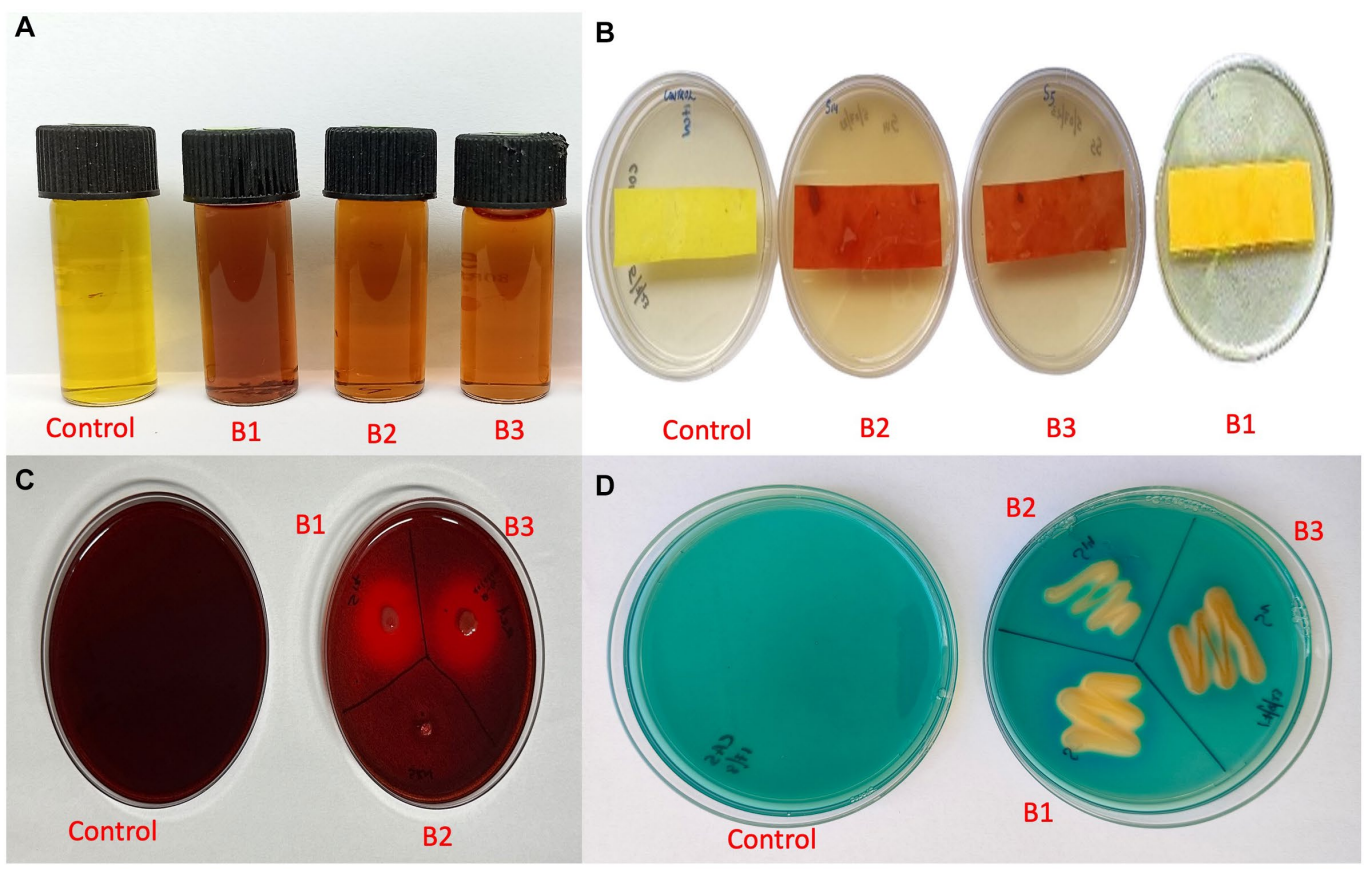
Qualitative assay for plant growth promotion traits **(A)** IAA production **(B)** HCN production **(C)** β-1-4-glucanse activity **(D)** Siderophore production (B1: *Pseudomonas aeruginosa*; B2: *Pseudomonas mosselii*; B3: *Pseudomonas sichuanensis*).

The qualitative assay for IAA production revealed that all three isolates were able to produce IAA as they produced a color change from yellow to pink at different intensities. Isolate B1 exhibited maximum IAA production (Figure 8A). HCN production was exhibited by isolates B2 and B3, where the yellow picrate filter paper changed to reddish-brown (Figure 8B). The isolates B1 and B3 produced a halo around the bacterial growth on CMC agar plates, which indicated the production of β-1-4-glucanse (Figure 8C). All the isolates were positive for siderophore production, where they exhibited a color change in the blue CAS agar medium (Figure 8D; Supplementary Table S4).

## 4 Discussion

The current study encompasses the comparative analysis of the composition and function of bacterial rhizobiome of the resistant and susceptible *Piper* species to reveal its role in plant–pathogen interactions. Venn analysis of the ASVs shared among the wild-resistant and cultivated susceptible rhizobiome revealed compositional variation in the bacterial diversity (Figure 1C). It is driven by plant species differences even though a portion of the community retains the niche homeostasis (Shi et al., 2022). The higher bacterial diversity indices in the rhizobiome of the wild-resistant *P. colubrinum* compared to the *Piper nigrum* are noteworthy. The enriched microbial community diversity in resistant plants compared to susceptible plants has been discussed in earlier studies in tobacco (Shi et al., 2022) and tomato (Zhang et al., 2020). A study reported that the resistant cultivars had more bacterial operational taxonomic units (OTUs) as they exhibited higher Chao1 richness indices even though there were no significant differences in the alpha diversity between the resistant and susceptible cultivars (Wei et al., 2019). The alpha diversity was higher in the resistant rhizobiome of *P. colubrinum* compared to *P. nigrum* even though the differences were not significant (Figures 1A,B). Li et al. found that the rhizosphere microbial community significantly differs by distinctly clustering based on the resistance and susceptibility of the different wheat varieties to *Fusarium* head blight (Li et al., 2023a). The diversity, structure, and composition of bacterial communities in the rhizosphere varied among potato scab-resistant and susceptible plant species (Kopecky et al., 2019). The PCoA analysis conducted on the bacterial community among the bulk soil and the rhizobiome of the *Piper* species revealed that the bacterial communities primarily clustered by the soil compartments as well as the species (Figure 2). Few studies revealed that the rhizosphere and bulk soil bacterial microbiome cluster together, while the endophytic communities cluster distinctly as it is highly dependent on the plant species (Afzal et al., 2019; Tkacz et al., 2020; Dutta et al., 2021). The root endosphere communities clustered distinctly between the susceptible and resistant *Piper* species (Figure 2). This finding remains consistent with the findings of Li et al. who found that the endophytic bacterial communities of the resistant peach cultivars clustered differently (Li et al., 2019). A recent study that explored the temporal dynamics of the rhizo-microbiome in eggplant discussed that the host plant recruits a diverse disease-suppressive rhizo-microbiome diversity which in turn influences its disease resistance (Zhang et al., 2024). Our diversity analysis falls in line with these studies as we could find a distinct diversity pattern in the bacterial rhizobiome of the wild-resistant and cultivated susceptible *Piper* species.

Phylum *Proteobacteria* are associated with a multitude of plant-associated bacteria endowed with the ability to fix nitrogen, cycle nutrients, stimulate plant development, and antagonize several soil-borne plant pathogens (Inderbitzin et al., 2018; Aqueel et al., 2023). The prominence of *Proteobacteria* in *P. nigrum* rhizosphere was evident in a study conducted at Yok Don national park in the Central Highlands of Vietnam (Tran et al., 2022). Obieze et al. characterized the abundance of the phylum *Proteobacteria* in the bacterial rhizosphere communities of cultivated black pepper (Obieze et al., 2023). The rhizobiome of *P. nigrum* and *P. colubrinum* showed the presence of *Proteobacteria* as the top abundant phylum (Supplementary Table S3). Acuña et al. reported the presence of *Proteobacteria* as the most abundant bacterial phylum in the rhizosphere and root endophytic compartments of wheat (Acuña et al., 2023). We found that the higher abundance of Proteobacteria was realized in the rhizobiome of wild-resistant *P. colubrinum* which may indicate its role in disease resistance. *Firmicutes* and *Bacteroidota (Bacteroidetes)* were the other two dominant phyla of which *Bacteriodota* dominated the resistant rhizobiome. This is in line with the findings of Tang et al., in which they showed that the phylum *Bacteriodota* was associated with rhizosphere where there was reduced occurrence of fusarium wilt and was negatively correlated to disease incidence. This reveals its role in inducing soil-borne disease resistance to associated crop plants (Cheng et al., 2020; Tang et al., 2020). At the genus level, the bacterial diversity differences were evident and the prominent genera was *Pseudomonas*, especially in the root endosphere of *P. colubrinum* compared to *P. nigrum* (Figure 3). *Pseudomonas* has great significance as a plant root endophyte and it plays an important role in promoting plant health by stimulating plant growth and suppressing soil-borne pathogens (Lu et al., 2022; Qian et al., 2022). The relevance and abundance of *Pseudomonas* as a root endophyte in suppressing a soil-borne disease such as *Verticillium* wilt in cotton was discussed by Zeng et al. (2022). Endophytic *Pseudomonas* species from *Piper tuberculatum* was found antagonistic to the *Fusarium* root rot in black pepper (Da Costa Pereira et al., 2019). These studies support our findings on *Pseudomonas* as a dominant endophyte in the resistant *P. colubrinum* root endosphere which can be prospected as a potential biocontrol agent against the oomycete pathogen.

The phylogenetic distinctness and differential abundance analysis revealed other significantly different genera in the resistant *P. colubrinum* rhizobiome such as *Comamonas*, Var*iovorax* (root endosphere), and *Janthinobacterium* (rhizosphere) (Supplementary Figures S3, S4). A prior study reported the increased abundance of *Comamonas* associated with reduced prevalence of *Phytophthora* blight in sweet pepper discussing its role in the biocontrol of soil-borne plant pathogens (Zhang et al., 2019). Booth et al. showed the potential use of *Comamonas* as a biopesticide against oomycetes pathogens in crops such as tomato, cabbage, and chickpea (Booth et al., 2022). The higher abundance of *Comamonas* was also associated with the healthy root endosphere of *Nicotiana tabacum* plants compared to *Ralstonia solanacearum-*infected plants (Li et al., 2023b). The genera *Variovorax* was associated with plant endophytic communities with potential biocontrol against *Phytophthora infestans* in grape vines (Bruisson et al., 2019; Vaghari Souran et al., 2023). *Janthinobacterium* present in rhizosphere soil displayed broad antagonism against the soil-borne oomycete pathogen *Pythium ultimum* and other fungal pathogens *such as Rhizoctonia solani* and *Fusarium graminearum* (Yin et al., 2021) and is known to produce antifungal compounds such as janthinopolyenemycins (Anjum et al., 2018). In our findings, we observed the significant differential abundance of these anti-oomycete genera in the resistant rhizobiome which indicates their potential role in the pathogen resistance in *P. colubrinum.*

In general, KEGG: level 2 metabolic pathway abundance showed a high degree of variability among the bulk soil, rhizosphere, and endophytic compartments of *P. colubrinum* and *P. nigrum* (Supplementary Figure S5). The pathway category “Cellular community- prokaryotes” is majorly related to quorum sensing and biofilm formation which significantly contributes to the colonization and establishment of beneficial bacteria in the rhizosphere (Herrera et al., 2016) “Biosynthesis of other secondary metabolites” includes gene functions such as phenyl propanoid biosynthesis, and biosynthesis of many antimicrobial compounds. Many of these compounds have proven antifungal activity and are synthesized by beneficial bacteria such as *Pseudomonas* and *Janthinobacterium* (Anjum et al., 2018; Ackermann et al., 2021). Osmoprotectants are small organic compounds that aid in maintaining the cell turgor and safeguarding cellular structures during plant stress. The ATP-binding protein is important for the active transport of osmoprotectants into bacterial cells via the osmoprotectant transport mechanism (Mishra et al., 2022). Methyl-accepting chemotaxis proteins (MCPs) are essential sensory receptors in bacteria that enable them to recognize and react to changes in their surroundings. As a result of this, beneficial bacteria such as *Pseudomonas* can detect infections and react by either moving away from potentially hazardous circumstances or producing antimicrobial compounds that hinder the dissemination of the pathogen (Feng et al., 2021; Ogura et al., 2021). KOs such as chemotaxis proteins, osmoprotectant, and other transport systems showed higher abundance in the rhizobiome of resistant *P. colubrinum* compared to susceptible *P. nigrum* (Supplementary Figure S6). Yang et al. studied the potential of the indirect manipulation of the plant disease resistance by metabolites that trigger the interaction of disease-suppressive microbiota to suppress the soil-borne *Ralstonia solanacearum* (Yang et al., 2023). Hence, these plant-specific metabolites may have the potential to induce disease resistance by recruiting these beneficial microbiota. Our analysis of the functional potential of the rhizobiome of *P. colubrinum* and *P. nigrum* suggests plant species can modulate specific metabolic pathways and may lead to better survival and resistance against pathogens in *P. colubrinum* compared to *P. nigrum.*

This study is the first of its kind even though the culturable diversity is lower possibly due to the limitations in cultural conditions. Studies have recognized that the identification of bacteria through metagenomic screening does not essentially correlate with their cultivability (Tan et al., 2015; Tlou et al., 2024). However, in our study, the resistant *P. colubrinum* root endosphere harbored the dominant genus *Pseudomonas* as the most effective biocontrol agent against *P. capsici*, in line with the 16srRNA amplicon data. A previous report suggested that the *P. colubrinum* housed more antagonistic endophytic bacterial genera than that of *P. nigrum* (Kollakkodan et al., 2017). The diversity of *Pseudomonas* in the root endosphere of *Piper* species was previously discussed in a few studies (Da Costa Pereira et al., 2019; Ashajyothi et al., 2020). The phylogenetic distinctness suggests the possible role of this genera in plant disease resistance. The biocontrol potential and plant growth promotion trait of *Pseudomonas* has been proven in many studies as discussed earlier (Da Costa Pereira et al., 2019; Lu et al., 2022; Qian et al., 2022; Zeng et al., 2022). This study is a first step toward exploring the root microbiome diversity of resistant *Piper* species in order to explore the role of these beneficial bacteria in conferring resistance against *Phytophthora capsici*.

## 5 Conclusion

In this study, we characterized the bacterial rhizobiome of resistant *Piper colubrinum* and susceptible *Piper nigrum* using metagenomic sequencing of the 16S rRNA gene. We compared the bacterial rhizobiome in these two plant species to ascertain the differences in their community composition, diversity, and function. Our hypothesis was based on the assertion that disease resistance in *P. colubrinum* may be contributed by the host genome along with its microbiome. We found significant differences in the bacterial community composition between the two species. In *P. colubrinum* rhizobiome, genera such as *Pseudomonas, Janthinobacterium*, *Variovorax*, and *Comamonas* were found to be differentially abundant compared to *P. nigrum*. Functional prediction revealed the abundance of KEGG pathways and KOs, which specifically support the colonization and deployment of beneficial bacteria with biocontrol activity in *P. colubrinum* rhizobiome. Our study confirms that the susceptible (*P. nigrum*) and resistant (*P. colubrinum*) species could harbor distinct bacterial genera in their rhizobiome although growing in the same environmental conditions. These findings could probably indicate the significance of plant species in shaping its rhizobiome. The culturable diversity of the resistant root endosphere, which harbors efficient biocontrol agents such as *Pseudomonas*, strengthens our hypothesis about the possible role of root microbiome in conferring resistance against soil-borne pathogens. Furthermore, this study lays the groundwork for gaining mechanistic insights into the plant species specificity to the bacterial rhizobiome in the *Piper* genus. These findings can be further substantiated by assessing the potential of the culturable isolates for plant growth promotion and biocontrol traits. However, to obtain a broader pattern of the plant–rhizobiome interaction, studies should also investigate the other underexplored species. To expand upon this study, future research could identify keystone taxa for microbiome engineering that can supplement resistance breeding approaches. Ultimately, these efforts could facilitate sustainable agriculture through microbiome-based plant breeding to mitigate crop diseases.

## Data availability statement

The datasets presented in this study can be found in online repositories. The names of the repository/repositories and accession number(s) can be found at: https://www.ncbi.nlm.nih.gov/, PRJNA991100.

## Author contributions

AP: Conceptualization, Writing – review & editing, Data curation, Formal analysis, Methodology, Validation, Visualization, Writing – original draft. RS: Conceptualization, Data curation, Formal analysis, Methodology, Validation, Writing – review & editing. ES: Conceptualization, Writing – review & editing, Funding acquisition, Project administration, Supervision.

## Footnotes

^1^ https://blast.ncbi.nlm.nih.gov/

## Supplementary material

Figure S1 Sampling location (Site A and B) at Western Ghats, Kerala, India.

Figure S2 Field Sampling and collection.

Figure S3 LDA score assessments of the size of differentiation among bulk soil and the hosts (score threshold 3).

Figure S4 Differential abundance analysis of bacterial genera in (a)Rhizosphere and (b)Root Endosphere in Piper spp. (ANCOM-BC;*P<0.05) (PNRE: Piper nigrum root endosphere; PNRS: Piper nigrum rhizosphere soil; PCRS: Piper colubrinum rhizosphere soil; PCRE: Piper colubrinum root endosphere).

Figure S5 Heat map analysis of the Kyoto Encyclopaedia of Genes and Genomes (KEGG) pathways at LEVEL 2(BS: Bulk soil; PNRE: Piper nigrum root endosphere; PNRS: Piper nigrum rhizosphere soil; PCRS: Piper colubrinum rhizosphere soil; PCRE: Piper colubrinum root endosphere).

Figure S6 Heat map analysis of top 100 predicted Kyoto Encyclopaedia of Genes and Genomes (KEGG) functional orthologs (BS: Bulk soil; PNRE: Piper nigrum root endosphere; PNRS: Piper nigrum rhizosphere soil; PCRE: Piper colubrinum root endosphere; PCRS: Piper colubrinum rhizosphere soil).
