# Supplementary figures and images for: Next-generation sequencing-based comparative mapping and culture-based screening of bacterial rhizobiome in Phytophthora capsici-resistant and susceptible Piper species
Source: Front Microbiol. 2024 Sep 25;15:1458454. doi: 10.3389/fmicb.2024.1458454 (PMC11472852; doi:10.3389/fmicb.2024.1458454)

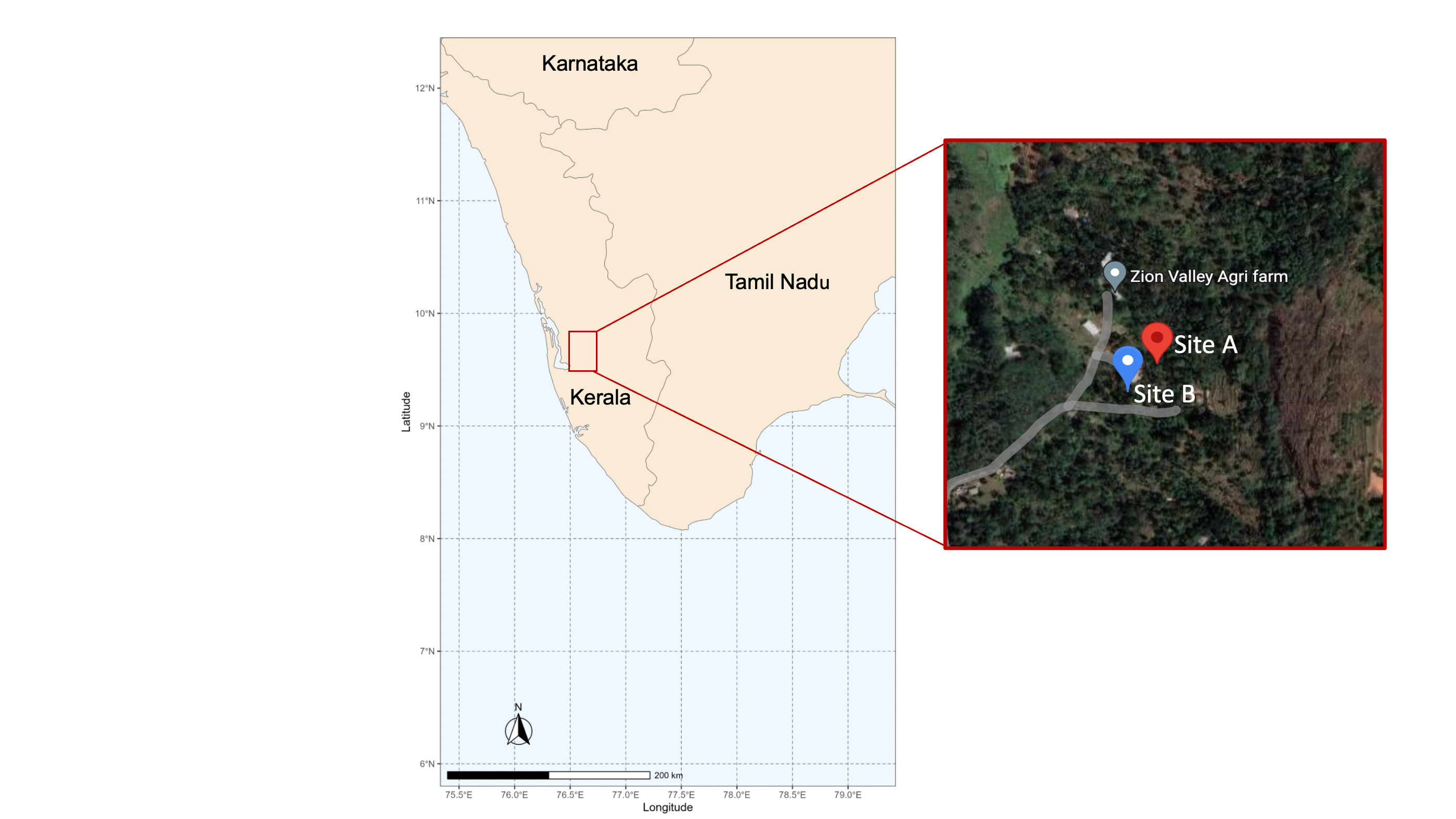

Supplement: Figure S1 — Sampling location (Site A and B) at Western Ghats, Kerala, India. [file Image_1.TIFF]

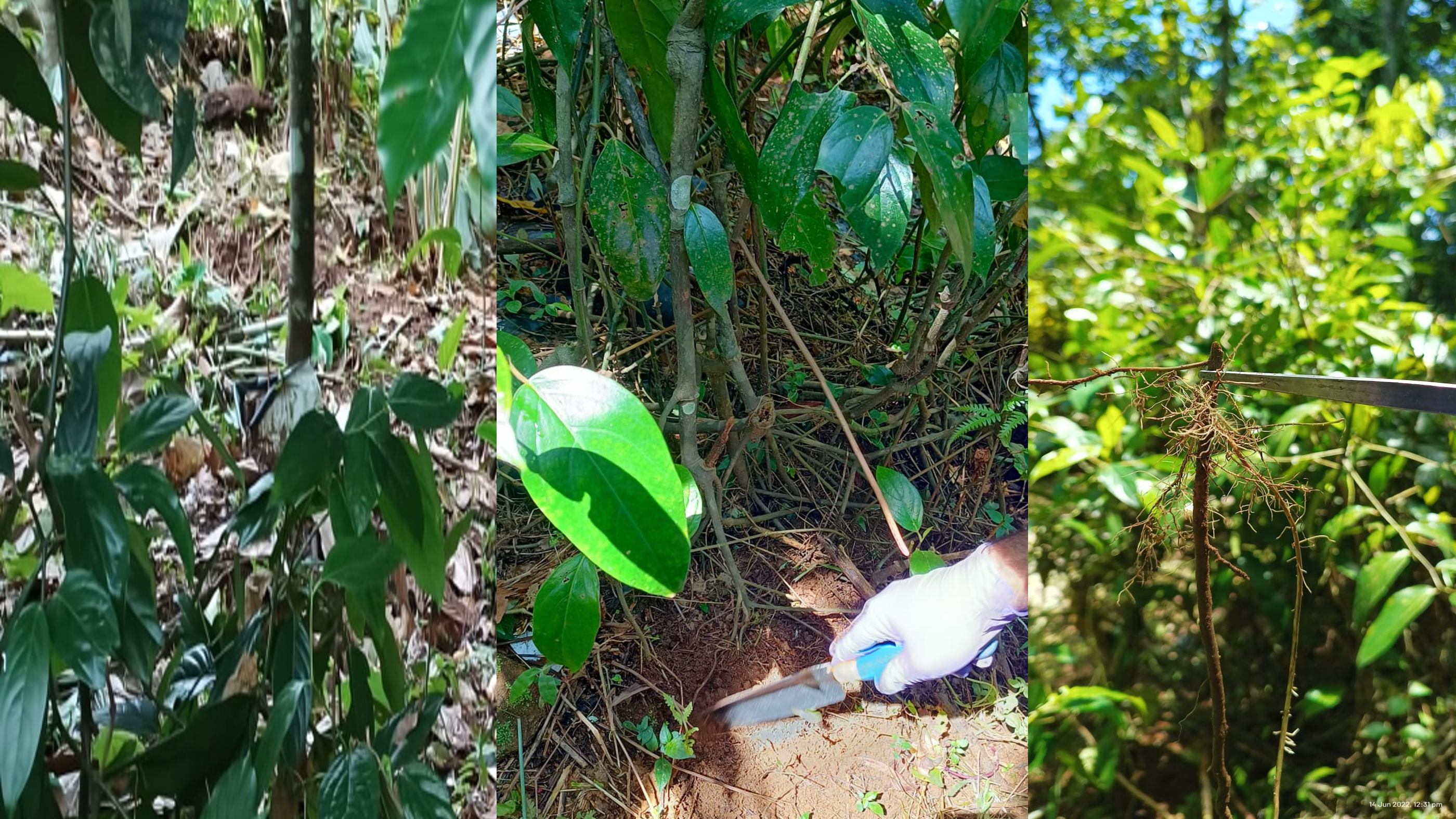

Supplement: Figure S2 — Field Sampling and collection. [file Image_2.TIFF]

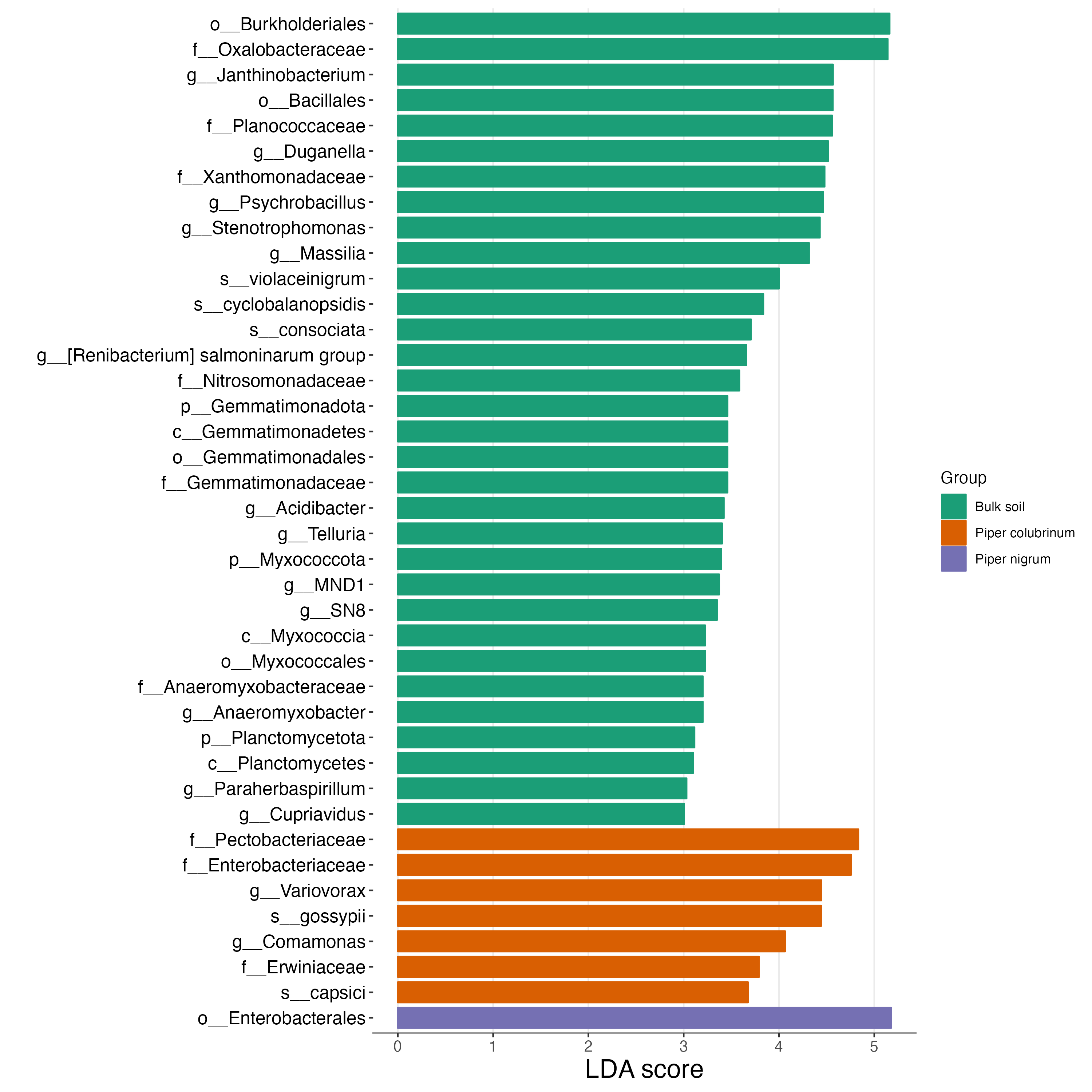

Supplement: Figure S3 — LDA score assessments of the size of differentiation among bulk soil and the hosts (score threshold 3). [file Image_3.TIFF]

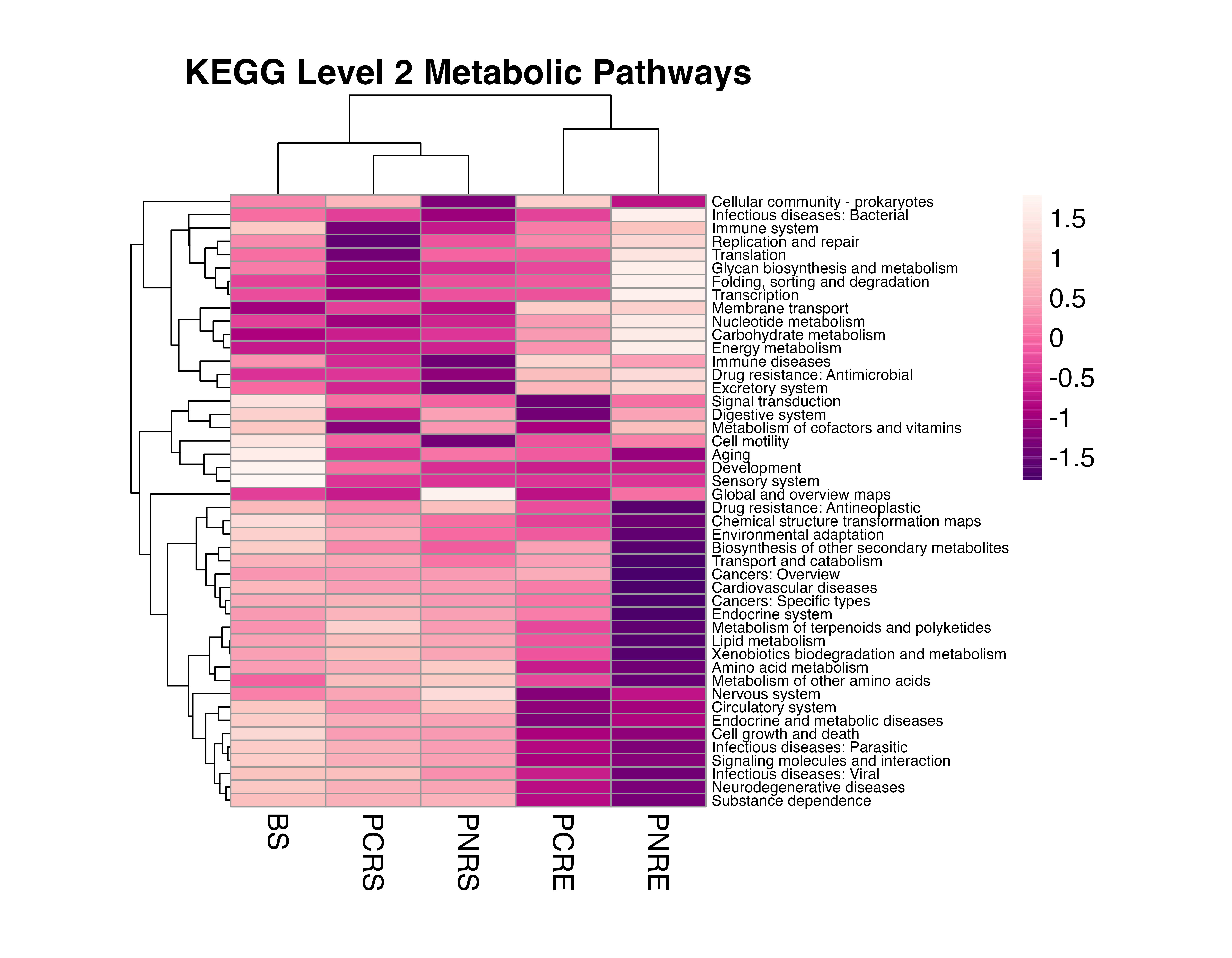

Supplement: Figure S5 — Heat map analysis of the Kyoto Encyclopaedia of Genes and Genomes (KEGG) pathways at LEVEL 2(BS: Bulk soil; PNRE: Piper nigrum root endosphere; PNRS: Piper nigrum rhizosphere soil; PCRS: Piper colubrinum rhizosphere soil; PCRE: Piper colubrinum root endosphere). [file Image_5.JPEG]
