# Supplementary material for: Next-generation sequencing-based comparative mapping and culture-based screening of bacterial rhizobiome in Phytophthora capsici-resistant and susceptible Piper species
Source: Front Microbiol. 2024 Sep 25;15:1458454. doi: 10.3389/fmicb.2024.1458454 (PMC11472852; doi:10.3389/fmicb.2024.1458454)
Supplement: Supplementary file 6 [file Table_1.DOCX]

**Table S1**: Sampling scheme (BS:Bulk soil;PNRE:*Piper nigrum* root endosphere;PNRS: *Piper nigrum* rhizosphere soil;PCRS: *Piper colubrinum* rhizosphere soil;PCRE:*Piper colubrinum* root endosphere)

| **SOURCE** | **Bulk soil (BS)** | | ***Piper colubrinum*** | | ***Piper nigrum*** | |
| --- | --- | --- | --- | --- | --- | --- |
| **SITE** | **A** | **B** | **A** | **B** | **A** | **B** |
|  | BSA1 | BSB1 | Rhizosphere soil (**PCRS**) | | Rhizosphere soil (**PNRS**) | |
|  | BSA2 | BSB2 | PCSA1 | PCSB1 | PNSA1 | PNSB1 |
|  | BSA3 | BSB3 | PCSA2 | PCSB2 | PNSA2 | PNSB2 |
|  |  |  | PCSA3 | PCSB3 | PNSA3 | PNSB3 |
|  |  |  | Root (**PCRE**) | | Root (**PNRE**) | |
|  |  |  | PCRA1 | PCRB1 | PNRA1 | PNRB1 |
|  |  |  | PCRA2 | PCRB2 | PNRA2 | PNRB2 |
|  |  |  | PCRA3 | PCRB3 | PNRA3 | PNRB3 |
