# Supplementary material for: Next-generation sequencing-based comparative mapping and culture-based screening of bacterial rhizobiome in Phytophthora capsici-resistant and susceptible Piper species
Source: Front Microbiol. 2024 Sep 25;15:1458454. doi: 10.3389/fmicb.2024.1458454 (PMC11472852; doi:10.3389/fmicb.2024.1458454)
Supplement: Supplementary file 7 [file Table_2.DOCX]

| **Comparison** | **Measure** | **Group** | **P.unadj** | **P.adj** | **Significance** |
| --- | --- | --- | --- | --- | --- |
| BS - PCRE | Observed | BS | 0.1320 | 0.2855 | ns |
| BS - PCRS | Observed | BS | 0.0050 | 0.0286 | * |
| BS - PNRE | Observed | BS | 0.0022 | 0.0247 | * |
| BS - PNRS | Observed | BS | 0.0043 | 0.0286 | * |
| PCRE - PCRS | Observed | PCRE | 0.4704 | 0.5967 | ns |
| PCRE - PNRE | Observed | PCRE | 0.1797 | 0.2933 | ns |
| PCRE - PNRS | Observed | PCRE | 1.0000 | 1.0000 | ns |
| PCRS - PNRE | Observed | PCRS | 0.4704 | 0.5967 | ns |
| PCRS - PNRS | Observed | PNRS | 0.1727 | 0.2933 | ns |
| PNRE - PNRS | Observed | PNRS | 0.0152 | 0.0606 | ns |
| BS - PCRE | Chao1 | BS | 0.0931 | 0.2402 | ns |
| BS - PCRS | Chao1 | BS | 0.0022 | 0.0247 | * |
| BS - PNRE | Chao1 | BS | 0.0022 | 0.0247 | * |
| BS - PNRS | Chao1 | BS | 0.0043 | 0.0286 | * |
| PCRE - PCRS | Chao1 | PCRE | 0.3939 | 0.5628 | ns |
| PCRE - PNRE | Chao1 | PCRE | 0.1797 | 0.2933 | ns |
| PCRE - PNRS | Chao1 | PCRE | 1.0000 | 1.0000 | ns |
| PCRS - PNRE | Chao1 | PCRS | 0.4848 | 0.5967 | ns |
| PCRS - PNRS | Chao1 | PNRS | 0.0931 | 0.2402 | ns |
| PNRE - PNRS | Chao1 | PNRS | 0.0161 | 0.0614 | ns |
| BS - PCRE | ACE | BS | 0.0931 | 0.2402 | ns |
| BS - PCRS | ACE | BS | 0.0022 | 0.0247 | * |
| BS - PNRE | ACE | BS | 0.0022 | 0.0247 | * |
| BS - PNRS | ACE | BS | 0.0043 | 0.0286 | * |
| PCRE - PCRS | ACE | PCRE | 0.3939 | 0.5628 | ns |
| PCRE - PNRE | ACE | PCRE | 0.1797 | 0.2933 | ns |
| PCRE - PNRS | ACE | PCRE | 1.0000 | 1.0000 | ns |
| PCRS - PNRE | ACE | PCRS | 0.4848 | 0.5967 | ns |
| PCRS - PNRS | ACE | PNRS | 0.0931 | 0.2402 | ns |
| PNRE - PNRS | ACE | PNRS | 0.0152 | 0.0606 | ns |
| BS - PCRE | Shannon | BS | 0.3095 | 0.4952 | ns |
| BS - PCRS | Shannon | BS | 0.0260 | 0.0903 | ns |
| BS - PNRE | Shannon | BS | 0.0087 | 0.0462 | * |
| BS - PNRS | Shannon | BS | 0.0152 | 0.0606 | ns |
| PCRE - PCRS | Shannon | PCRE | 0.4848 | 0.5967 | ns |
| PCRE - PNRE | Shannon | PCRE | 0.0649 | 0.2078 | ns |
| PCRE - PNRS | Shannon | PCRE | 0.3939 | 0.5628 | ns |
| PCRS - PNRE | Shannon | PCRS | 0.3939 | 0.5628 | ns |
| PCRS - PNRS | Shannon | PCRS | 0.6991 | 0.7558 | ns |
| PNRE - PNRS | Shannon | PNRS | 0.1320 | 0.2855 | ns |
| BS - PCRE | Simpson | BS | 0.5887 | 0.6826 | ns |
| BS - PCRS | Simpson | BS | 0.4848 | 0.5967 | ns |
| BS - PNRE | Simpson | BS | 0.1797 | 0.2933 | ns |
| BS - PNRS | Simpson | BS | 0.1320 | 0.2855 | ns |
| PCRE - PCRS | Simpson | PCRS | 0.6991 | 0.7558 | ns |
| PCRE - PNRE | Simpson | PCRE | 0.0931 | 0.2402 | ns |
| PCRE - PNRS | Simpson | PCRE | 0.1797 | 0.2933 | ns |
| PCRS - PNRE | Simpson | PCRS | 0.3939 | 0.5628 | ns |
| PCRS - PNRS | Simpson | PCRS | 0.5887 | 0.6826 | ns |
| PNRE - PNRS | Simpson | PNRS | 0.6991 | 0.7558 | ns |
| BS - PCRE | InvSimpson | BS | 0.5887 | 0.6826 | ns |
| BS - PCRS | InvSimpson | BS | 0.4848 | 0.5967 | ns |
| BS - PNRE | InvSimpson | BS | 0.1797 | 0.2933 | ns |
| BS - PNRS | InvSimpson | BS | 0.1320 | 0.2855 | ns |
| PCRE - PCRS | InvSimpson | PCRS | 0.6991 | 0.7558 | ns |
| PCRE - PNRE | InvSimpson | PCRE | 0.0931 | 0.2402 | ns |
| PCRE - PNRS | InvSimpson | PCRE | 0.1797 | 0.2933 | ns |
| PCRS - PNRE | InvSimpson | PCRS | 0.3939 | 0.5628 | ns |
| PCRS - PNRS | InvSimpson | PCRS | 0.5887 | 0.6826 | ns |
| PNRE - PNRS | InvSimpson | PNRS | 0.6991 | 0.7558 | ns |
| BS - PCRE | Fisher | BS | 0.1320 | 0.2855 | ns |
| BS - PCRS | Fisher | BS | 0.0050 | 0.0286 | * |
| BS - PNRE | Fisher | BS | 0.0022 | 0.0247 | * |
| BS - PNRS | Fisher | BS | 0.0043 | 0.0286 | * |
| PCRE - PCRS | Fisher | PCRE | 0.4704 | 0.5967 | ns |
| PCRE - PNRE | Fisher | PCRE | 0.1797 | 0.2933 | ns |
| PCRE - PNRS | Fisher | PCRE | 1.0000 | 1.0000 | ns |
| PCRS - PNRE | Fisher | PCRS | 0.4704 | 0.5967 | ns |
| PCRS - PNRS | Fisher | PNRS | 0.1727 | 0.2933 | ns |
| PNRE - PNRS | Fisher | PNRS | 0.0152 | 0.0606 | ns |

**Table S2**: Alpha diversity indices of bacterial amplicon sequence variants (ASVs);(**P*<0.05)

(BS:Bulk soil;PNRE:*Piper nigrum* root endosphere;PNRS: *Piper nigrum* rhizosphere soil;PCRS: *Piper colubrinum* rhizosphere soil;PCRE:*Piper colubrinum* root endosphere)
