# Supplementary material for: Next-generation sequencing-based comparative mapping and culture-based screening of bacterial rhizobiome in Phytophthora capsici-resistant and susceptible Piper species
Source: Front Microbiol. 2024 Sep 25;15:1458454. doi: 10.3389/fmicb.2024.1458454 (PMC11472852; doi:10.3389/fmicb.2024.1458454)
Supplement: Supplementary file 8 [file Table_3.DOCX]

**Table S3**: Taxonomic composition at phylum, class and genus level in bulk soil and host rhizobiome (BS:Bulk soil;PNRE:*Piper nigrum* root endosphere;PNRS: *Piper nigrum* rhizosphere soil;PCRS: *Piper colubrinum* rhizosphere soil;PCRE:*Piper colubrinum* root endosphere)

| **Sample groups** | **Taxonomy** | **Abundance** | **SD** | **SE** | **All_mean_abund** |
| --- | --- | --- | --- | --- | --- |
| **BS** | **Phylum** |  |  |  |  |
|  | *Proteobacteria* | 84.9187 | 8.2747 | 3.3781 | 0.8745 |
|  | *Firmicutes* | 7.7141 | 8.1422 | 3.3240 | 0.0795 |
|  | *Bacteroidota* | 4.9050 | 2.1156 | 0.8637 | 0.0330 |
|  | *Gemmatimonadota* | 0.6034 | 0.3079 | 0.1257 | 0.0015 |
|  | *Myxococcota* | 0.5056 | 0.2381 | 0.0972 | 0.0014 |
|  | **Class** |  |  |  |  |
|  | *Gammaproteobacteria* | 83.7877 | 8.7198 | 3.5598 | 0.8625 |
|  | *Bacilli* | 7.7043 | 8.1462 | 3.3257 | 0.0338 |
|  | *Bacteroidia* | 4.9050 | 2.1156 | 0.8637 | 0.0330 |
|  | *Alphaproteobacteria* | 1.1310 | 0.5994 | 0.2447 | 0.0120 |
|  | *Gemmatimonadetes* | 0.6034 | 0.3079 | 0.1257 | 0.0015 |
|  | **Genus** |  |  |  |  |
|  | *Pseudomonas* | 29.1074 | 18.6973 | 7.6331 | 0.3025 |
|  | *Janthinobacterium* | 8.0317 | 7.4228 | 3.0304 | 0.0332 |
|  | *Duganella* | 7.6188 | 2.9930 | 1.2219 | 0.0271 |
|  | *Stenotrophomonas* | 6.6955 | 3.5074 | 1.4319 | 0.0254 |
|  | *Psychrobacillus* | 5.8259 | 6.3301 | 2.5842 | 0.0124 |
| **PCRS** | **Phylum** |  |  |  |  |
|  | *Proteobacteria* | 92.8648 | 1.1240 | 0.4589 | 0.8745 |
|  | *Bacteroidota* | 6.2924 | 1.2326 | 0.5032 | 0.0330 |
|  | *Actinobacteriota* | 0.3737 | 0.4847 | 0.1979 | 0.0049 |
|  | *Verrucomicrobiota* | 0.2614 | 0.3419 | 0.1396 | 0.0030 |
|  | *Firmicutes* | 0.1490 | 0.1982 | 0.0809 | 0.0795 |
|  | **Class** |  |  |  |  |
|  | *Gammaproteobacteria* | 92.1173 | 1.0380 | 0.4238 | 0.8625 |
|  | *Bacteroidia* | 6.2924 | 1.2326 | 0.5032 | 0.0330 |
|  | *Alphaproteobacteria* | 0.7475 | 0.3206 | 0.1309 | 0.0120 |
|  | *Actinobacteria* | 0.3737 | 0.4847 | 0.1979 | 0.0045 |
|  | *Verrucomicrobiae* | 0.2614 | 0.3419 | 0.1396 | 0.0030 |
|  | **Genus** |  |  |  |  |
|  | *Pseudomonas* | 44.7237 | 12.9735 | 5.2964 | 0.3025 |
|  | *Oxalicibacterium* | 6.6295 | 5.7730 | 2.3568 | 0.0189 |
|  | *Variovorax* | 5.5865 | 1.9043 | 0.7774 | 0.0395 |
|  | *Janthinobacterium* | 5.4961 | 3.5264 | 1.4397 | 0.0332 |
|  | *Comamonas* | 3.5639 | 5.3286 | 2.1754 | 0.0161 |
| **PCRE** | **Phylum** |  |  |  |  |
|  | *Proteobacteria* | 93.3167 | 1.0487 | 0.4281 | 0.8745 |
|  | *Firmicutes* | 4.5264 | 2.6556 | 1.0841 | 0.0795 |
|  | *Bacteroidota* | 1.0797 | 1.2986 | 0.5302 | 0.0330 |
|  | *Actinobacteriota* | 0.8208 | 1.0618 | 0.4335 | 0.0049 |
|  | *Verrucomicrobiota* | 0.0708 | 0.0826 | 0.0337 | 0.0030 |
|  | **Class** |  |  |  |  |
|  | *Gammaproteobacteria* | 91.7680 | 1.2887 | 0.5261 | 0.8625 |
|  | *Negativicutes* | 2.5404 | 2.3106 | 0.9433 | 0.0394 |
|  | *Alphaproteobacteria* | 1.5487 | 1.0697 | 0.4367 | 0.0120 |
|  | *Bacteroidia* | 1.0797 | 1.2986 | 0.5302 | 0.0330 |
|  | *Bacilli* | 1.0040 | 0.8297 | 0.3387 | 0.0338 |
|  | **Genus** |  |  |  |  |
|  | *Pectobacterium* | 23.0764 | 20.8916 | 8.5290 | 0.0727 |
|  | *Pseudomonas* | 12.7559 | 7.0848 | 2.8923 | 0.3025 |
|  | *Variovorax* | 8.0561 | 5.4811 | 2.2377 | 0.0395 |
|  | *Enterobacter* | 4.2992 | 4.7391 | 1.9347 | 0.0280 |
|  | *Aeromonas* | 3.7203 | 3.1030 | 1.2668 | 0.0693 |
| **PNRS** | **Phylum** |  |  |  |  |
|  | *Proteobacteria* | 89.6771 | 10.2250 | 4.1743 | 0.8745 |
|  | *Firmicutes* | 4.8195 | 8.0968 | 3.3055 | 0.0795 |
|  | *Bacteroidota* | 3.9841 | 2.2761 | 0.9292 | 0.0330 |
|  | *Verrucomicrobiota* | 0.7914 | 1.0837 | 0.4424 | 0.0030 |
|  | *Actinobacteriota* | 0.3444 | 0.7378 | 0.3012 | 0.0049 |
|  | **Class** |  |  |  |  |
|  | *Gammaproteobacteria* | 88.5974 | 10.3769 | 4.2363 | 0.8625 |
|  | *Bacilli* | 4.6876 | 8.1781 | 3.3387 | 0.0338 |
|  | *Bacteroidia* | 3.9841 | 2.2761 | 0.9292 | 0.0330 |
|  | *Alphaproteobacteria* | 1.0797 | 0.5158 | 0.2106 | 0.0120 |
|  | *Verrucomicrobiae* | 0.7841 | 1.0671 | 0.4356 | 0.0030 |
|  | **Genus** |  |  |  |  |
|  | *Pseudomonas* | 55.3373 | 9.5780 | 3.9102 | 0.3025 |
|  | *Lactococcus* | 3.8790 | 7.4573 | 3.0444 | 0.0098 |
|  | *Variovorax* | 3.7911 | 2.2335 | 0.9118 | 0.0395 |
|  | *Aeromonas* | 3.4564 | 3.5117 | 1.4336 | 0.0693 |
|  | *Stenotrophomonas* | 2.8336 | 2.0249 | 0.8266 | 0.0254 |
| **PNRE** | **Phylum** |  |  |  |  |
|  | *Proteobacteria* | 76.4840 | 16.5392 | 6.7521 | 0.8745 |
|  | *Firmicutes* | 22.5658 | 17.4606 | 7.1283 | 0.0795 |
|  | *Bacteroidota* | 0.4470 | 0.5354 | 0.2186 | 0.0049 |
|  | *Actinobacteriota* | 0.2150 | 0.3188 | 0.1301 | 0.0330 |
|  | *Verrucomicrobiota* | 0.1637 | 0.1571 | 0.0642 | 0.0030 |
|  | **Class** |  |  |  |  |
|  | *Gammaproteobacteria* | 75.0037 | 14.9216 | 6.0917 | 0.8625 |
|  | *Negativicutes* | 16.9769 | 13.8085 | 5.6373 | 0.0394 |
|  | *Bacilli* | 3.3954 | 4.0974 | 1.6728 | 0.0338 |
|  | *Clostridia* | 2.1936 | 2.1000 | 0.8573 | 0.0064 |
|  | *Alphaproteobacteria* | 1.4803 | 1.8811 | 0.7680 | 0.0120 |
|  | **Genus** |  |  |  |  |
|  | *Aeromonas* | 25.5704 | 15.1423 | 6.1818 | 0.0693 |
|  | *Pelosinus* | 16.9061 | 13.8053 | 5.6360 | 0.0390 |
|  | *Pseudomonas* | 9.3287 | 6.2861 | 2.5663 | 0.3025 |
|  | *Pectobacterium* | 8.5129 | 16.8997 | 6.8993 | 0.0727 |
|  | *Enterobacter* | 7.1132 | 5.1712 | 2.1111 | 0.0280 |
