# Supplementary material for: Next-generation sequencing-based comparative mapping and culture-based screening of bacterial rhizobiome in Phytophthora capsici-resistant and susceptible Piper species
Source: Front Microbiol. 2024 Sep 25;15:1458454. doi: 10.3389/fmicb.2024.1458454 (PMC11472852; doi:10.3389/fmicb.2024.1458454)
Supplement: Supplementary file 9 [file Table_4.DOCX]

**Table S4:** Qualitative analysis of plant growth promotion potential of endophytic bacterial isolates **(**B1**:** *Pseudomonas aeruginosa*; B2: *Pseudomonas mosselii*; B3: *Pseudomonas sichuanensis*)

| **ISOLATES** | **IAA PRODUCTION** | **HCN PRODUCTION** | **β – GLUCANASE PRODUCTION** | **SIDEROPHORE PRODCUTION** |
| --- | --- | --- | --- | --- |
| B1 | **++** | **-** | **+** | **+** |
| B2 | **-** | **+** | **-** | **+** |
| B3 | **+** | **+** | **+** | **+** |
